# Supplementary material for: Detailed Phenotyping and Therapeutic Strategies for Intronic ABCA4 Variants in Stargardt Disease
Source: Mol Ther Nucleic Acids. 2020 Jun 12;21:412–27. doi: 10.1016/j.omtn.2020.06.007 (PMC7352060; doi:10.1016/j.omtn.2020.06.007)
Supplement: Document S1. Figures S1–S8 [file mmc1.pdf]

OMTN, Volume *21*

Supplemental Information

Detailed Phenotyping and Therapeutic Strategies for Intronic *ABCA4* Variants in Stargardt Disease

Mubeen Khan, Gavin Arno, Ana Fakin, David A. Parfitt, Patty P.A. Dhooge, Silvia Albert, Nathalie M. Bax, Lonneke Duijkers, Michael Niblock, Kwan L. Hau, Edward Bloch, Elena R. Schiff, Davide Piccolo, Michael C. Hogden, Carel B. Hoyng, Andrew R. Webster, Frans P.M. Cremers, Michael E. Cheetham, Alejandro Garanto, and Rob W.J. Collin

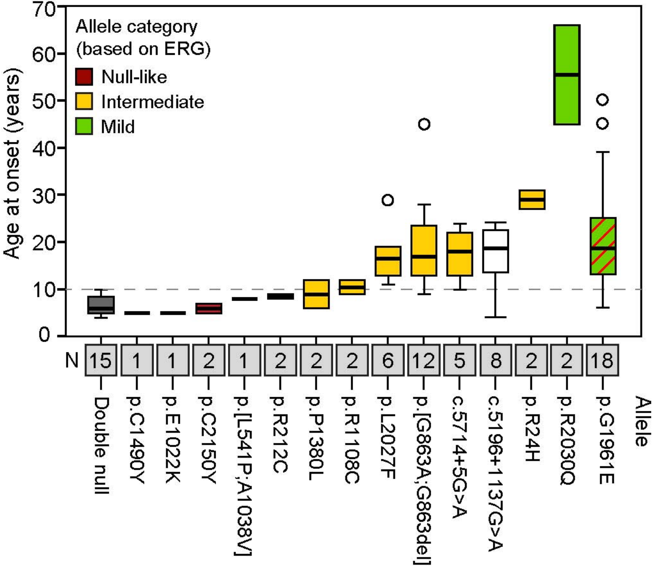

Allele category
(based on ERG)
Null-like
Intermediate
Mild
Age at onset (years)
0
10
20
30
40
50
60
70
N
15
1
1
2
1
2
2
2
6
12
5
8
2
2
18
Double null
p.C1490Y
p.E1022K
p.C2150Y
p.[L541P;A1038V]
p.R212C
p.P1380L
p.R1108C
p.L2027F
p.[G863A;G863del]
c.5714+5G>A
c.5196+1137G>A
p.R24H
p.R2030Q
p.G1961E
Allele

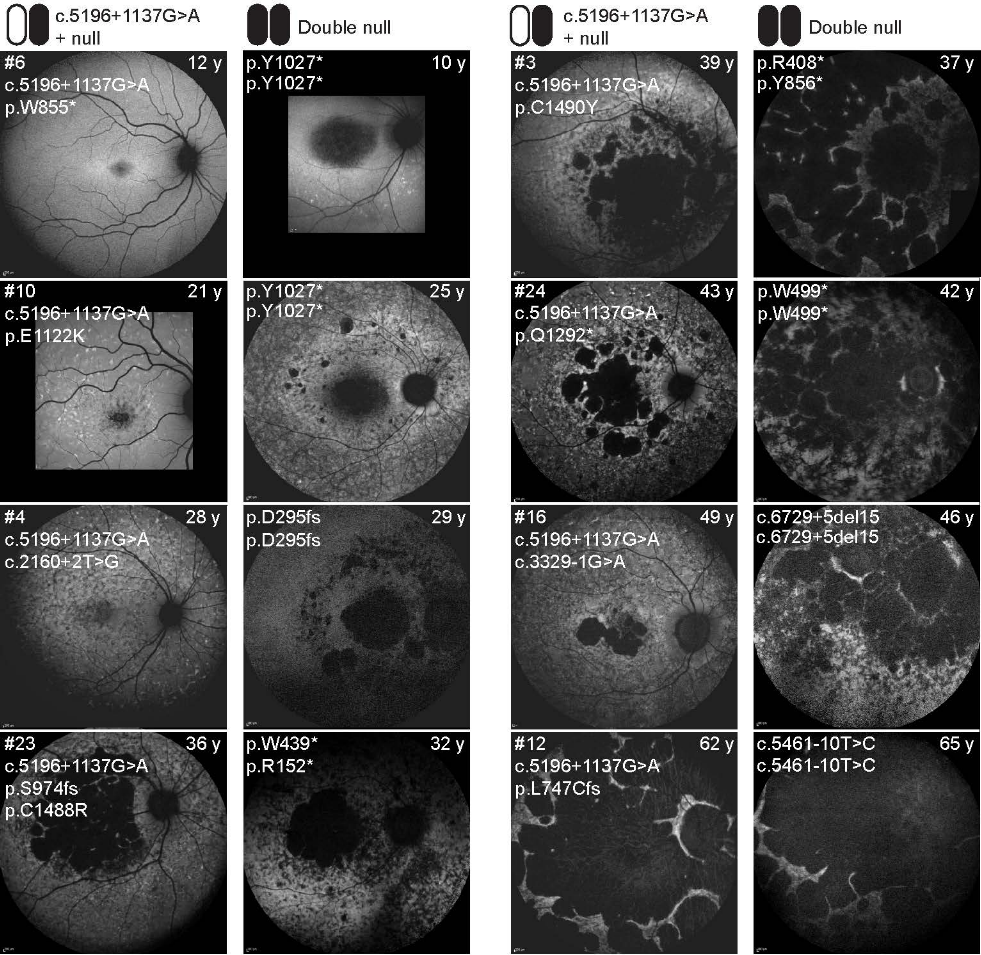

c.5196+1137G>A
+ null
Double null
c.5196+1137G>A
+ null
Double null
#6
12 y
c.5196+1137G>A
p.W855*
p.Y1027*
10 y
p.Y1027*
#3
39 y
c.5196+1137G>A
p.C1490Y
p.R408*
37 y
p.Y856*
#10
21 y
c.5196+1137G>A
p.E1122K
p.Y1027*
25 y
p.Y1027*
#24
43 y
c.5196+1137G>A
p.Q1292*
p.W499*
42 y
p.W499*
#4
28 y
c.5196+1137G>A
c.2160+2T>G
p.D295fs
29 y
p.D295fs
#16
49 y
c.5196+1137G>A
c.3329-1G>A
c.6729+5del15
46 y
c.6729+5del15
#23
36 y
c.5196+1137G>A
p.S974fs
p.C1488R
p.W439*
32 y
p.R152*
#12
62 y
c.5196+1137G>A
p.L747Cfs
c.5461-10T>C
65 y
c.5461-10T>C

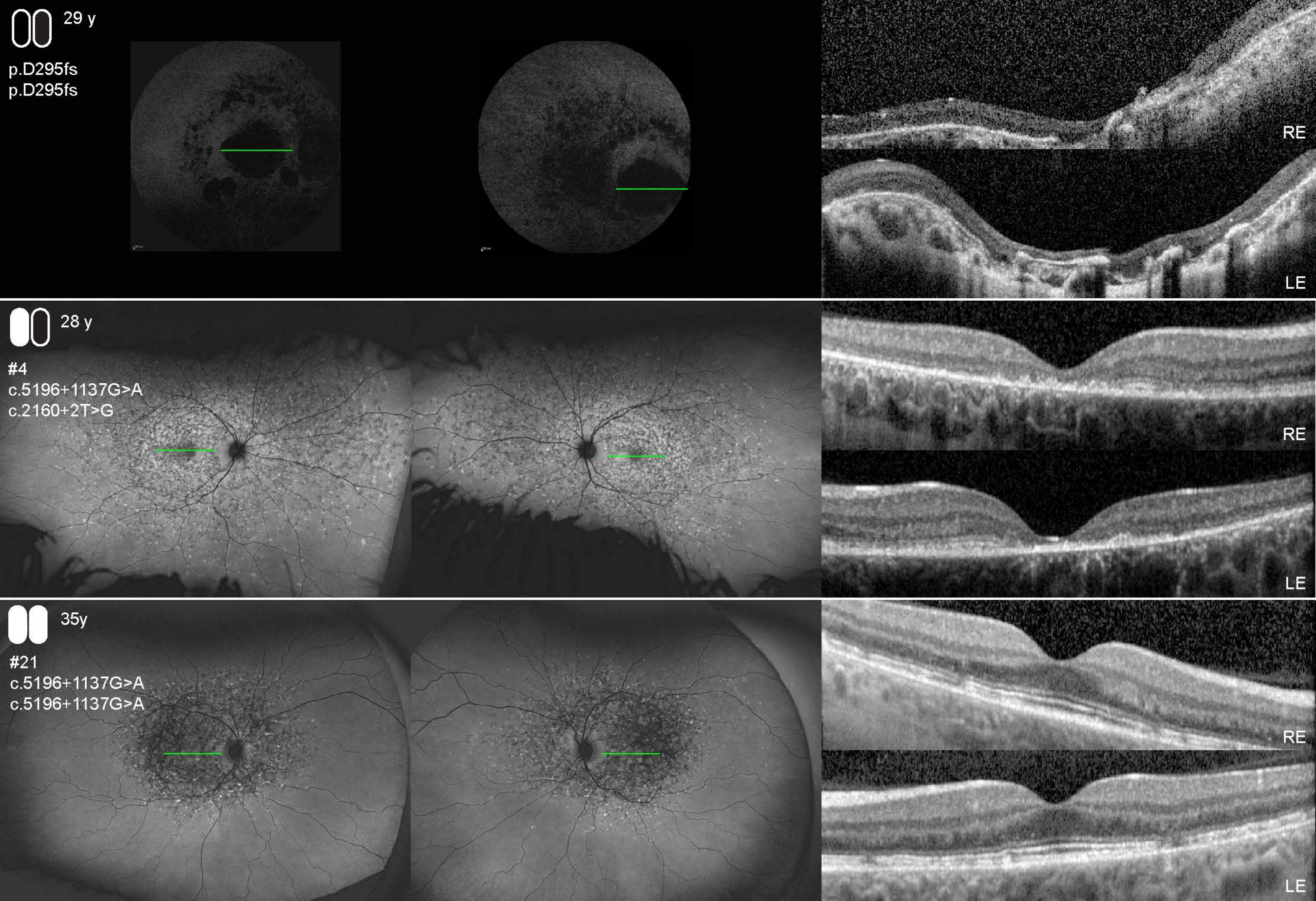

29 y
p.D295fs
p.D295fs
RE
LE
28 y
#4
c.5196+1137G>A
c.2160+2T>G
RE
LE
35y
#21
c.5196+1137G>A
c.5196+1137G>A
RE
LE

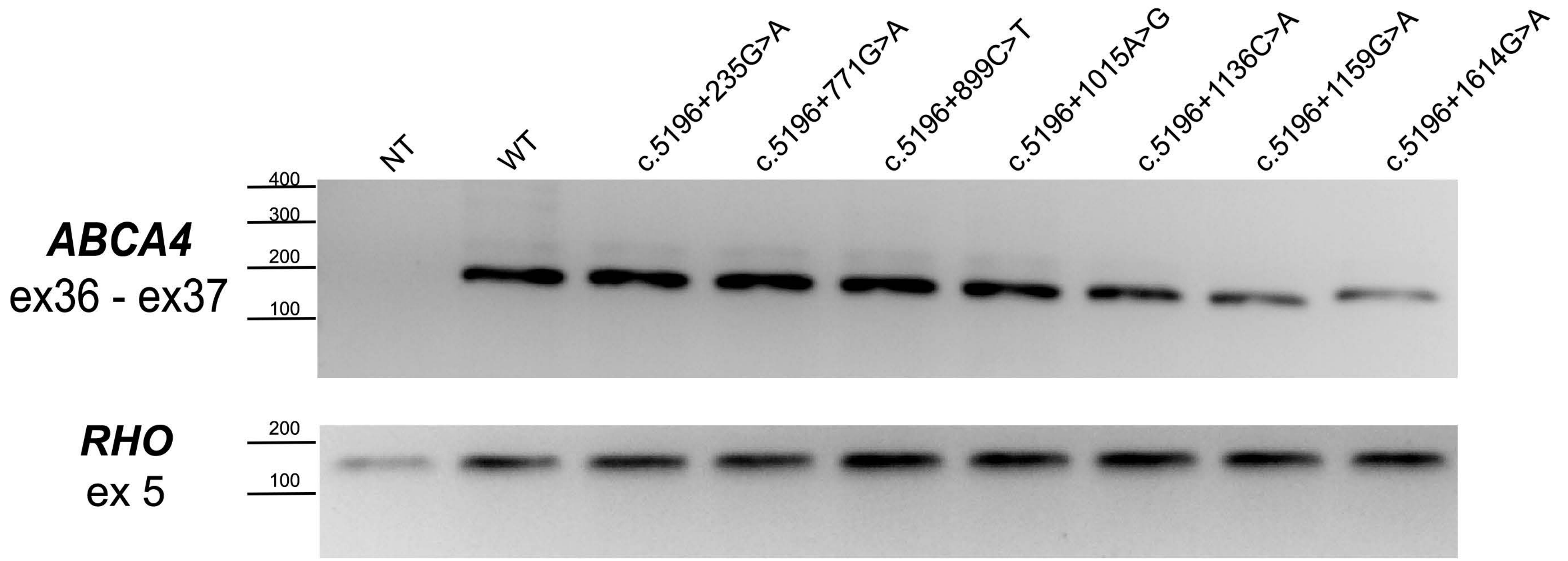

NT
WT
c.5196+235G>A
c.5196+771G>A
c.5196+899C>T
c.5196+1015A>G
c.5196+1136C>A
c.5196+1159G>A
c.5196+1614G>A
ABCA4
ex36 - ex37
400
300
200
100
RHO
ex 5
200
100

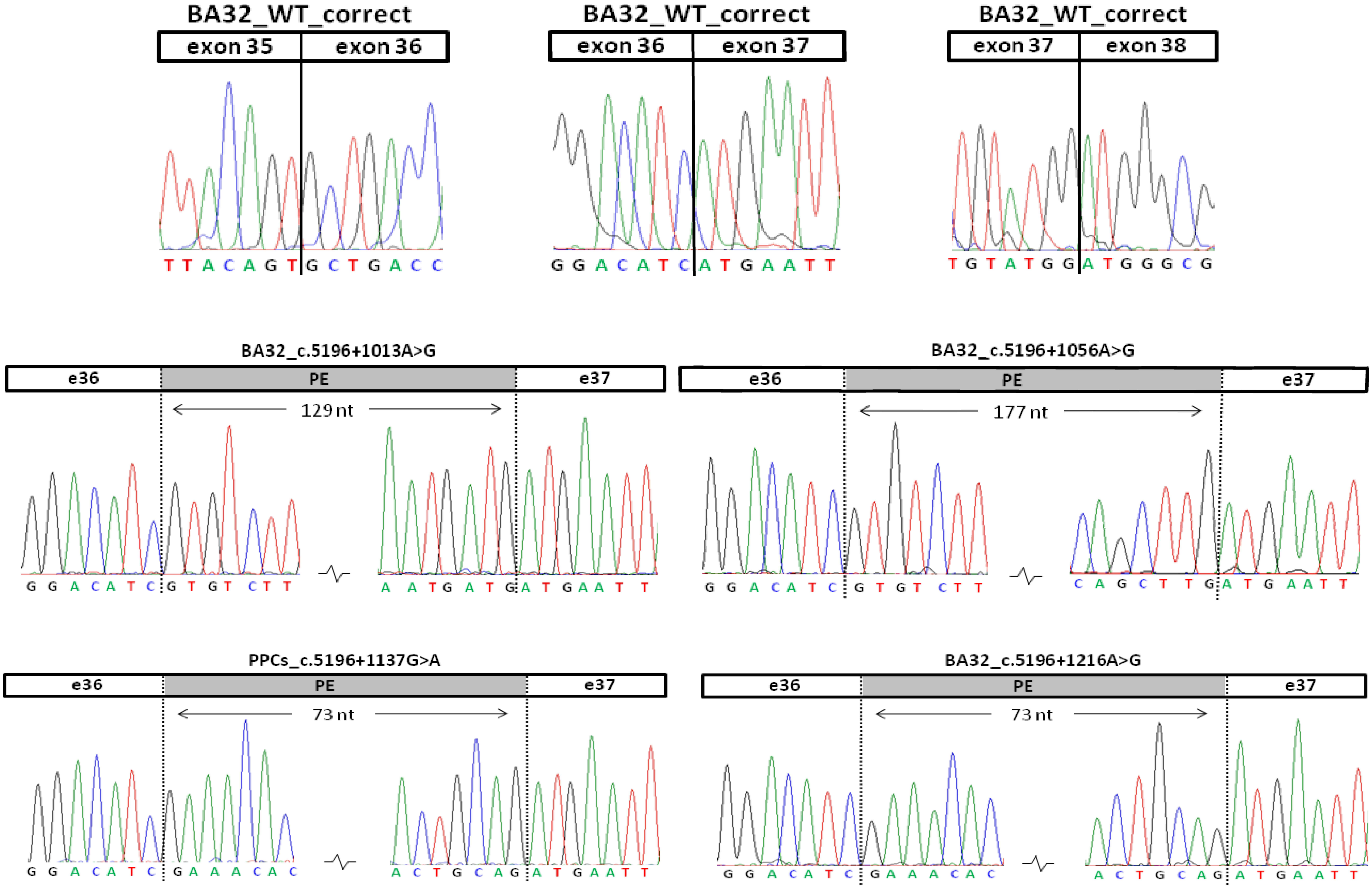
BA32_WT_correct
exon 35
exon 36
T T A C A G T G C T G A C C
BA32_WT_correct
exon 36
exon 37
G G A C A T C A T G A A T T
BA32_WT_correct
exon 37
exon 38
T G T A T G G A T G G G C G
BA32_c.5196+1013A>G
e36
PE
e37
129 nt
G G A C A T C G T G T C T T
A A T G A T G A T G A A T T
BA32_c.5196+1056A>G
e36
PE
e37
177 nt
G G A C A T C G T G T C T T
C A G C T T G A T G A A T T
PPCs_c.5196+1137G>A
e36
PE
e37
73 nt
G G A C A T C G A A A C A C
A C T G C A G A T G A A T T
BA32_c.5196+1216A>G
e36
PE
e37
73 nt
G G A C A T C G A A A C A C
A C T G C A G A T G A A T T

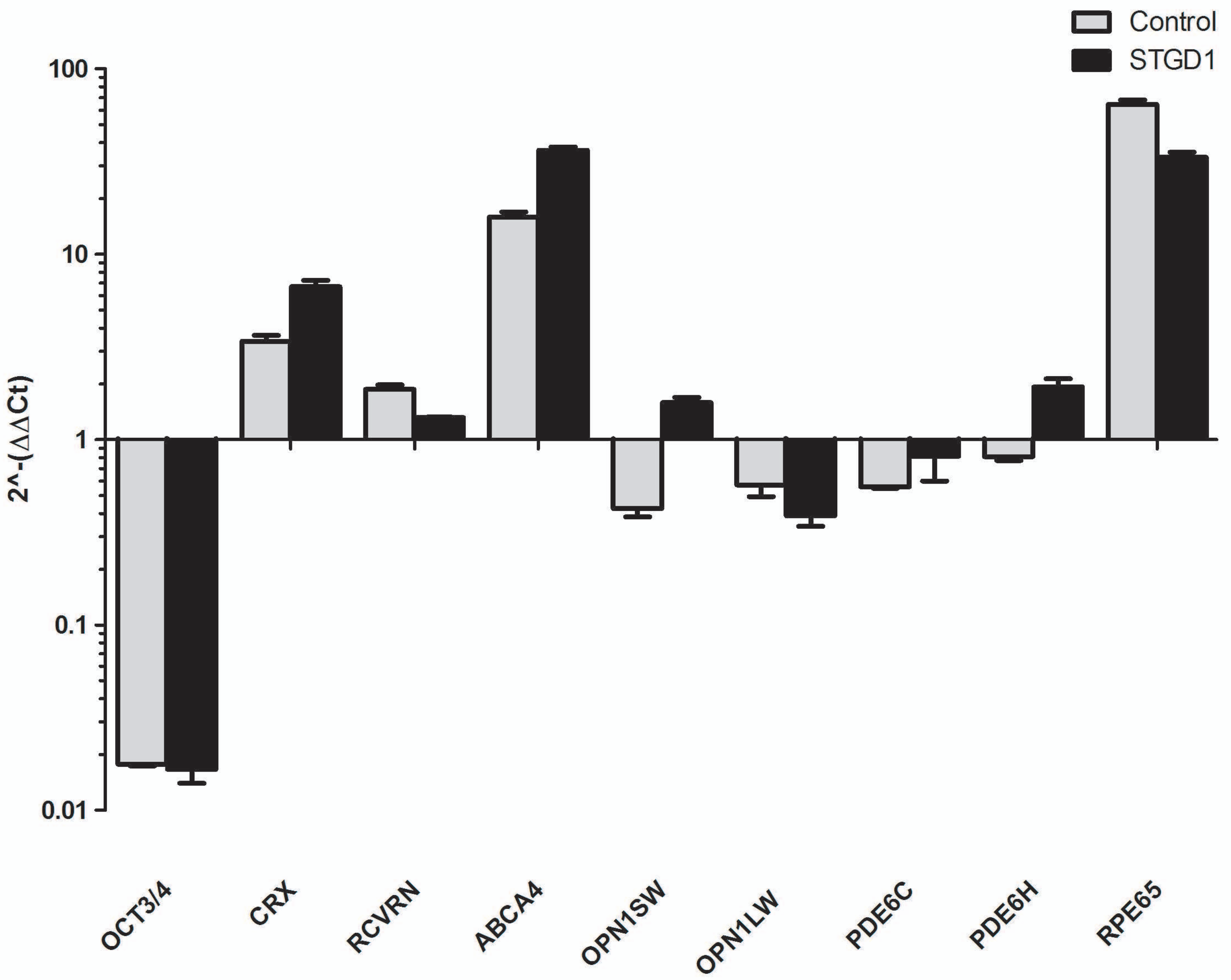

Control
STGD1
100
10
1
0.1
0.01
2^-(ΔΔCt)
OCT3/4
CRX
RCVRN
ABCA4
OPN1SW
OPN1LW
PDE6C
PDE6H
RPE65

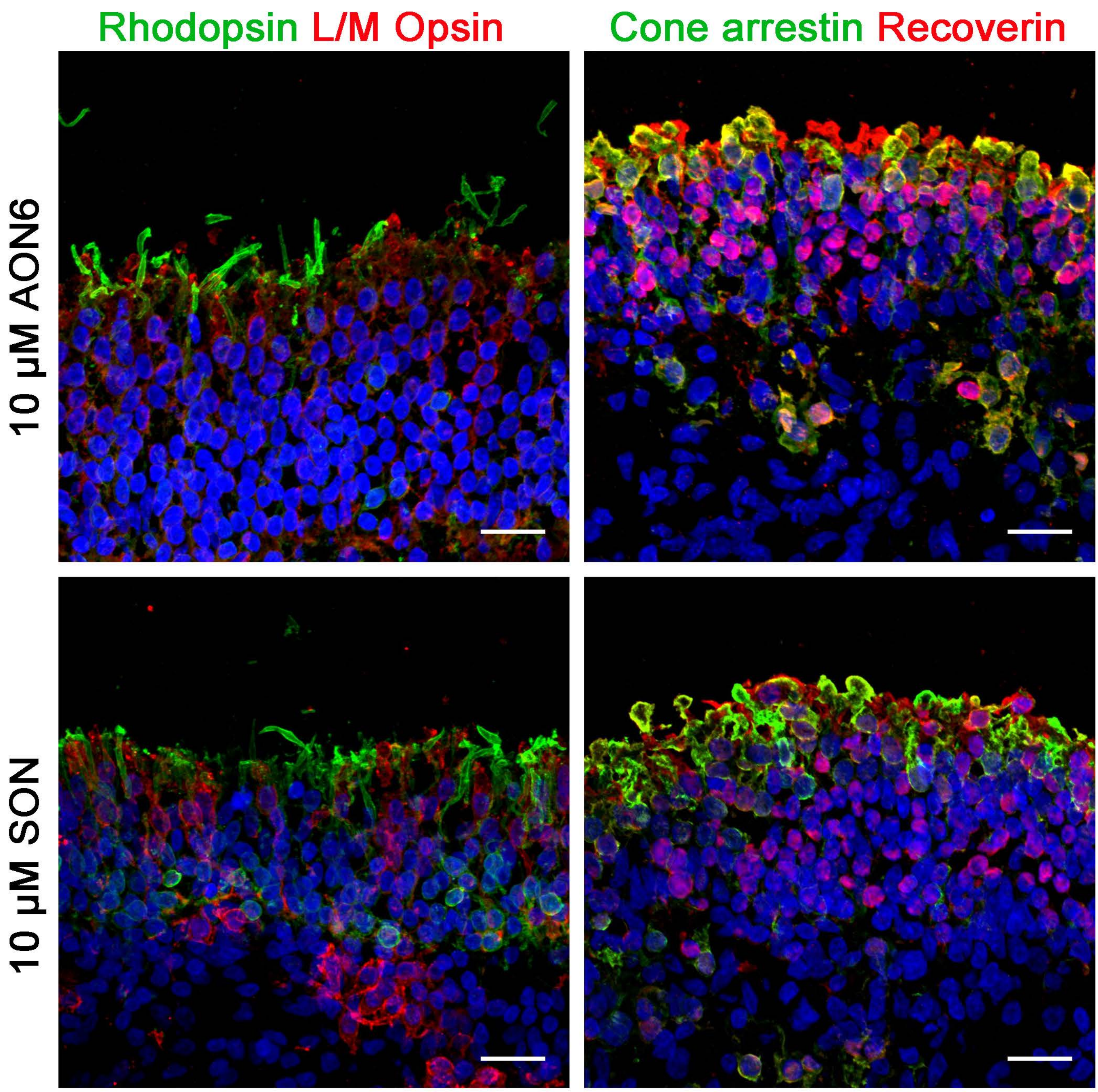
Rhodopsin L/M Opsin
Cone arrestin Recoverin
10 μM AON6
10 μM SON

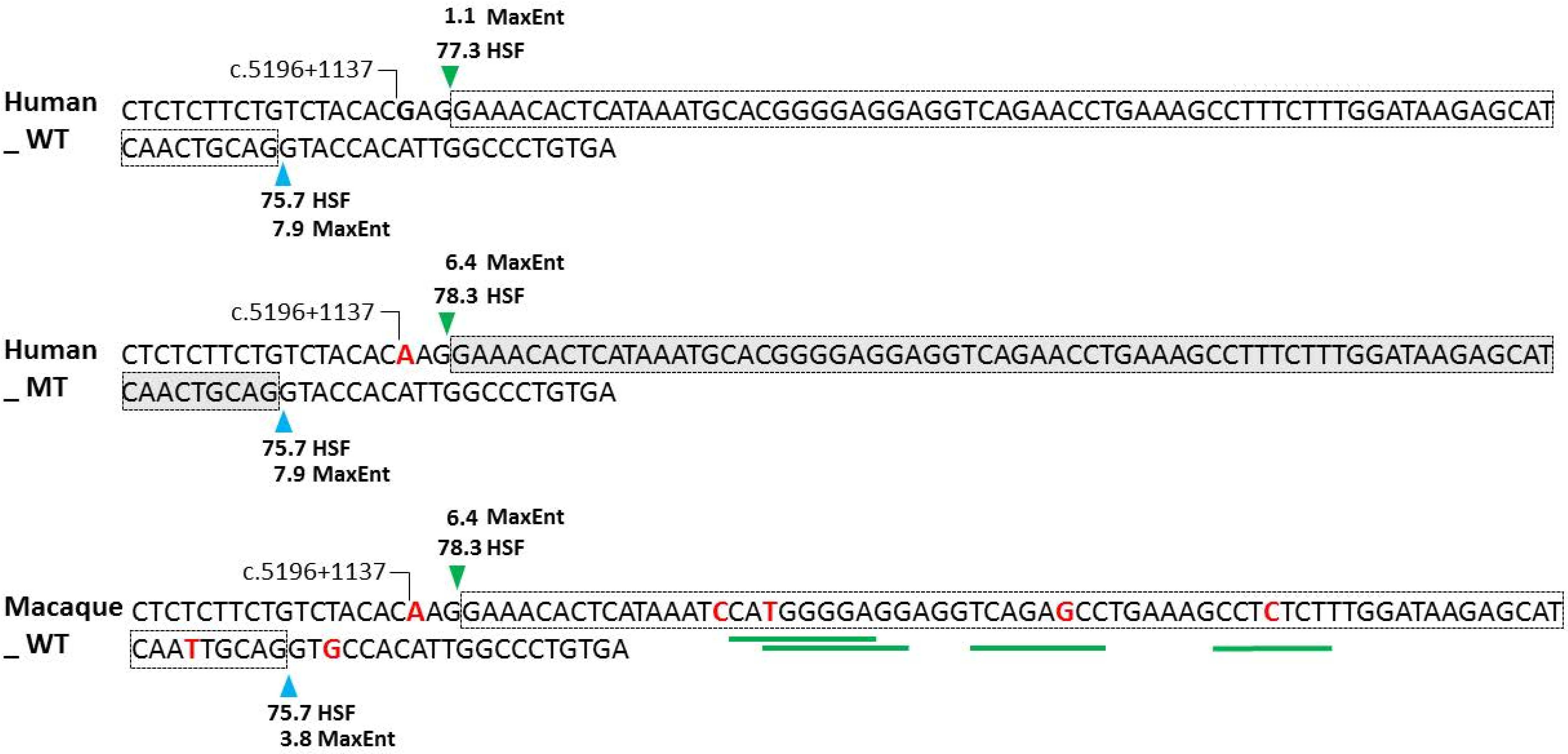
1.1 MaxEnt
77.3 HSF
c.5196+1137
Human _ WT
CTCTCTTCTGTCTACACGAGGAAACACTCATAAATGCACGGGGAGGAGGTCAGAACCTGAAAGCCTTTCTTTGGATAAGAGCAT
CAACTGCAGGTACCACATTGGCCCTGTGA
75.7 HSF
7.9 MaxEnt
6.4 MaxEnt
78.3 HSF
c.5196+1137
Human _ MT
CTCTCTTCTGTCTACACAAGGAAACACTCATAAATGCACGGGGAGGAGGTCAGAACCTGAAAGCCTTTCTTTGGATAAGAGCAT
CAACTGCAGGTACCACATTGGCCCTGTGA
75.7 HSF
7.9 MaxEnt
6.4 MaxEnt
78.3 HSF
c.5196+1137
Macaque _ WT
CTCTCTTCTGTCTACACAAGGAAACACTCATAAATCCATGGGGAGGAGGTCAGAGCCTGAAAGCCTCTCTTTGGATAAGAGCAT
CAATTGCAGGTGCCACATTGGCCCTGTGA
75.7 HSF
3.8 MaxEnt

Supplemental Figure Legends

Supplemental Figure S1

Distribution of the ages at onset associated with different *ABCA4* alleles in *trans* with null alleles. The figure is modified from Fakin et al,[7] with the addition of c.5196+1137G>A. Different categories of alleles were determined based on full-field ERG. Allele p.G1961E had a specific preponderance to foveal damage, reflected by a relatively early onset of visual symptoms. Dashed line marks the 95% confidence interval of the patients harboring two null alleles.

Supplemental Figure S2

Fundus autofluorescence images of patients harboring c.5196+1137G>A in *trans* with null alleles and age-matched patients harboring two null alleles. Patient ID and genotypes are noted in the top left corner; age is noted in the top right corner of each image.

Supplemental Figure S3

FAF and OCT of a patient harboring two null alleles (top), a patient harboring c.5196+1137G>A in *trans* with a null allele (middle) and a patient homozygous for c.5196+1137G>A patient of similar ages. Note the increasingly milder phenotype in the presence of c.5196+1137G>A alleles.

Supplemental Figure S4

Sanger sequencing electropherograms for four causal *ABCA4* variants in intron 36 and correct transcript are given. Sequence traces for variants tested in *in vitro* splice assays are labeled as BA32_respective variant, whereas results obtained from photoreceptor progenitor cells (PPCs) are labelled as PPCs variants. PE, pseudoexon; nt, nucleotide.

Supplemental Figure S5

Seven *ABCA4* variants in intron 36 were tested in wild-type (WT) construct BA32 (*ABCA4* exons 35 - exon 38). RT-PCR was performed by using exonic (ex) primers in exon 36 and 37 of *ABCA4* for WT and all the variants. Rhodopsin (*RHO*) exon 5 amplification was used as a transfection and loading control.

Supplemental Figure S6

Gene expression profile of photoreceptor precursor cells (PPCs) derived from control (grey bars) and c.5196+1137G>A STGD1 (black bars) iPSCs. Expression levels were assessed by qPCR, normalized to GUSB expression and compared to the expression profile of day 0 iPSCs (undifferentiated). Pluripotency marker expression (*OCT3/4*) was reduced, while the expression of the photoreceptor precursor marker (*CRX*) was increased. The differentiation into RPE-like cells is shown by the increased expression of *RPE65*, while PPCs (especially STGD1 cells) showed some expression of early (*RCVRN*, *OPN1SW*) and late (*OPN1LW, PDE6C or PDE6H*). The expression of *ABCA4* was highly increased in all cell lines. The results are shown as the mean ± SD of two experimental replicates with three technical replicates each.

Supplemental Figure S7

Immunocytochemistry of retinal organoids treated with either AON or SON. Sections were stained with antibodies for rhodopsin (green, left panels), L/M opsin (red, left panels), cone arrestin (green, right panels) and recoverin (red, right panels). Nuclei were stained with DAPI in blue. Scale bar: 20 µM.

Supplemental Figure S8

Splice-site predictions are given for human wild type (WT) and mutant (MT) sequence as well as for the macaque WT. Strength of the WT and MT splice acceptor site (SAS) and splice donor site (SDS) were calculated using Human splicing Finder (HSF) and MaxEnt splicing prediction tools, by taking 20 nt upstream and downstream of the 73-nt pseudo-exon (PE). The PE found in the human mutant sequence is highlighted in grey. Nucleotides that are different among human WT/MT and macaque WT are shown in red. Green bars indicate the newly created splicing silencer elements due to the difference in human and macaque sequences. Green and blue rectangles indicate the SAS and SDS respectively.
