# Supplementary material for: Detailed Phenotyping and Therapeutic Strategies for Intronic ABCA4 Variants in Stargardt Disease
Source: Mol Ther Nucleic Acids. 2020 Jun 12;21:412–27. doi: 10.1016/j.omtn.2020.06.007 (PMC7352060; doi:10.1016/j.omtn.2020.06.007)
Supplement: Document S2. Article plus Supplemental Information [file mmc6.pdf]

Molecular Therapy
Nucleic Acids
Original Article

Detailed Phenotyping and Therapeutic Strategies for Intronic *ABCA4* Variants in Stargardt Disease

Mubeen Khan,[1,2,9] Gavin Arno,[3,4,5,9] Ana Fakin,[3,4,6,9] David A. Parfitt,[3] Patty P.A. Dhooge,[2,7] Silvia Albert,[1,2] Nathalie M. Bax,[2,7] Lonneke Duijkers,[1] Michael Niblock,[3] Kwan L. Hau,[3] Edward Bloch,[3,4] Elena R. Schiff,[3] Davide Piccolo,[3] Michael C. Hogden,[8] Carel B. Hoyng,[2,7] Andrew R. Webster,[3,4] Frans P.M. Cremers,[1,2] Michael E. Cheetham,[3] Alejandro Garanto,[1,2,10] and Rob W.J. Collin[1,2,10]

[1]Department of Human Genetics, Radboud University Medical Center, Nijmegen, the Netherlands; [2]Donders Institute for Brain, Cognition and Behavior, Radboud University Medical Center, Nijmegen, the Netherlands; [3]UCL Institute for Ophthalmology, London, UK; [4]Moorfields Eye Hospital, London, UK; [5]Great Ormond Street Hospital for Children, London, UK; [6]Eye Hospital, University Medical Centre Ljubljana, Ljubljana, Slovenia; [7]Department of Ophthalmology, Radboud University Medical Center, Nijmegen, the Netherlands; [8]Princess Alexandra Hospital, Brisbane, QLD, Australia

Stargardt disease is a progressive retinal disorder caused by bi-allelic mutations in the *ABCA4* gene that encodes the ATP-binding cassette, subfamily A, member 4 transporter protein. Over the past few years, we and others have identified several pathogenic variants that reside within the introns of *ABCA4*, including a recurrent variant in intron 36 (c.5196+1137G>A) of which the pathogenicity so far remained controversial. Detailed clinical characterization of this variant confirmed its pathogenic nature, and classified it as an allele of intermediate severity. Moreover, we discovered several additional *ABCA4* variants clustering in intron 36. Several of these variants resulted in aberrant splicing of *ABCA4*, i.e., the inclusion of pseudoexons, while the splicing defects caused by the recurrent c.5196+1137G>A variant strongly increased upon differentiation of patient-derived induced pluripotent stem cells into retina-like cells. Finally, all splicing defects could be rescued by the administration of antisense oligonucleotides that were designed to specifically block the pseudoexon insertion, including rescue in 3D retinal organoids harboring the c.5196+1137G>A variant. Our data illustrate the importance of intronic variants in *ABCA4* and expand the therapeutic possibilities for overcoming splicing defects in Stargardt disease.

INTRODUCTION

Stargardt disease (STGD1), also often referred to as *ABCA4*-associated retinopathy, is a progressive disorder of the retina, initially characterized by a loss of central vision.[1] It is the most common form of inherited macular dystrophy and is believed to affect ~1 in 10,000 individuals worldwide.[1] There is a high degree of clinical heterogeneity in many patients, resulting in severe visual impairment or even complete blindness, due to death of the photoreceptors and the retinal pigment epithelium (RPE) cells.

ABCA4-associated retinopathy is caused by bi-allelic variants in *ABCA4*, a 50-exon gene that codes for the ATP-binding cassette, subfamily A, member 4, a transmembrane protein of 2,273 aa (ABCA4, previously known as ABCR).[2] The protein belongs to the superfamily of ATP-binding cassette transporters,[2] and within retinal photoreceptor cells, it is located at the rim of the outer segment disks in rods and lamellae in cones, where it acts as a flippase facilitating the active transport of potentially toxic retinoids.[3] Dysfunction or absence of ABCA4 leads to an accumulation of these toxic by-products of the visual cycle, commonly known as lipofuscin, in the RPE after outer segment phagocytosis. Another potential mechanism of disease is direct toxicity toward the cone photoreceptors due to their different anatomical structure.[4]

ABCA4 displays a tremendous allelic heterogeneity, with more than 1,000 pathogenic mutations identified to date (accessible in https://databases.lovd.nl/shared/variants/ABCA4/).[5] There is a strong correlation reported between the clinical phenotypic appearance and the severity of the *ABCA4* genotype in a given patient.[6,7] Indeed, variants that lead to complete loss of function of ABCA4 result in severe disease,[8] whereas other variants are very mild and, in some cases, even show reduced penetrance in the absence of other severe *cis*-acting *ABCA4* variants.[9]

Over the past decades, we and others have identified several *ABCA4* variants that are located outside the protein-coding exons of the gene.[10–16] The majority of these are deep-intronic pathogenic variants that result in the activation of a splice site within an intron leading to the insertion of an aberrant pseudoexon (PE) into the *ABCA4* pre-mRNA. Following splicing, these PEs often result in disruption of the reading frame and are expected to result in nonsense-mediated

Received 6 March 2020; accepted 9 June 2020;
https://doi.org/10.1016/j.omtn.2020.06.007.

[9]These authors contributed equally to this work.

[10]These authors contributed equally to this work.

Correspondence: Rob W.J. Collin, Department of Human Genetics (855), Radboud University Medical Center, Geert Grooteplein Zuid 10, 6525 GA, Nijmegen, the Netherlands.
E-mail: rob.collin@radboudumc.nl

 © 2020 The Authors.
This is an open access article under the CC BY license (http://creativecommons.org/licenses/by/4.0/).

decay (NMD) of the aberrant transcript.[17] Thus, these mutations can lead to reduced, or even the absence of, functional ABCA4 protein, dependent on the extent of PE inclusion and the degree of NMD. Intriguingly, the recognition of the PEs by the splicing machinery can vary significantly, in terms of both the quantity of PE inclusion[14] and tissue dependency.[10,18] In 2013, Braun and colleagues[19] were the first to report pathogenic deep-intronic *ABCA4* variants, including a change in intron 36 (c.5196+1137G>A, reported there as "V1") that was recurrently present in their primary as well as their validation cohort of patients with *ABCA4*-associated retinopathy. This variant and a second closely located variant (c.5196+1216C>A) each strengthened alternative splicing and inclusion of a 73-nt PE into a small fraction of *ABCA4* transcripts in keratinocytes derived from patients who showed a compound heterozygosity for one of these alleles.

The pathogenic mechanism of PE inclusion consequent upon deep-intronic variants has the potential to be highly amenable for splicing modulating therapeutic approaches using antisense oligonucleotides (AONs). These relatively small RNA molecules of 18–25 nt can bind complementarily to their target pre-mRNA, preventing the inclusion of the PE into the final mRNA transcript upon splicing.[20] AONs can be considered safe and well tolerated when delivered to the retina, as shown in the results of a recent phase I/II clinical trial for a recurrent deep-intronic variant in *CEP290*.[21]

In this study, we performed a detailed phenotypic characterization of a substantial cohort of STGD1 patients harboring the c.5196+1137G>A variant and predicted its severity based on the clinical presentation in patients who harbored a null variant on the other allele. In addition, we expanded the search for deep-intronic mutations in intron 36 and assessed their pathogenicity based on their ability to dysregulate *ABCA4* pre-mRNA splicing in both midigene splicing assays and patient-derived retina-like cells.[22] Finally, for four variants that cause splicing defects, these defects were rescued by the administration of AONs, thereby opening new avenues for treating selected subgroups of STGD1 patients.

RESULTS

Variant c.5196+1137G>A Is an Allele of Intermediate Severity

Phenotypic data of 25 patients harboring the c.5196+1137G>A mutation are shown in Table 1. The median age at onset was 24 years (range = 4–55 years), and the last exam was performed at a median age of 43 years (range = 12–72 years). The majority (92%; 23/25) exhibited fundus autofluorescence (FAF) abnormalities (hyper- or hypoautofluorescence) within and beyond the vascular arcades, while in 8% (2/25), the abnormalities were confined within the vascular arcades (Figure 1). Foveal photoreceptors were affected in the majority (21/25) of patients and were preserved (foveal sparing) in three. The median age at electroretinography (ERG) recording was 36 years (range: 15–68 years; n = 21). At that time, according to the previously established ERG classification,[23] 67% (14/21) of the patients were classified into group 1 (normal full-field ERG [ffERG]; ages 15–68 years), and 24% (5/21) were classified into group 3 (abnormal cone and rod function; ages 36–61 years) (Table 1). Two patients had normal photopic responses, while their scotopic responses were subnormal, which is a pattern not included in the previous classification. For the patients recorded at the same institute (Moorfields Eye Hospital, London, UK [MEH]; n = 16), cross-sectional ERG results were plotted against their age (Figure 2A).

To determine the severity of the c.5196+1137G>A mutation, a subgroup of eight patients harboring this allele *in trans* with a null or null-like allele (Table 2, indicated with # therein) was analyzed separately (Figures 1A and 2B). This allowed the assessment of the c.5196+1137G>A allele in isolation, an approach that has been used previously to determine the severity of *ABCA4* alleles.[7] The ERG amplitudes of these patients were significantly better than those of the "double null" patients (Figure 2B). The homozygous patient had normal ffERG; however, the amplitudes are not plotted on the chart, as the recording was done at a different institute. The distribution of ages at onset for the different genotypes is shown in Figure S1. The median age at onset of the patients harboring c.5196+1137G>A *in trans* with a null or null-like allele was significantly higher than that of the patients harboring two null alleles (19 years vs. 6 years, Mann-Whitney U test, $p < 0.01$). It was most comparable to previously defined alleles of intermediate severity, e.g., p.R24H, c.5714+5G>A and p.L2027F. The homozygous patient had disease onset at the age of 35 years.

The FAF images of the eight selected patients were also arranged by age and compared with age-matched patients harboring two null alleles. In the early stages, the macular area was notably more preserved in c.5196+1137G>A patients. In later stages, when the atrophy expanded outside the imaged area, the differences were less obvious. None of these patients exhibited foveal sparing (Figure 1A; Figure S2). The homozygous patient had a notably different phenotype. FAF showed widespread abnormalities at the posterior pole and beyond the vascular arcades, while the optical coherence tomography (OCT) revealed preserved photoreceptors across the whole macula, including those in the fovea (Figure 1; Figure S3). The patient also had well preserved visual acuity (6/9 on both eyes at age 35; Table 1), while none of the patients harboring c.5196+1137G>A *in trans* with a loss-of-function allele had visual acuity above 6/24 (ages 12–62).

Variant c.5196+1137G>A Occurs on Different Haplotypes

Haplotype analysis was performed using variants located within the *ABCA4* locus and is summarized in Table 2 and Table S1. For some of the individuals carrying the c.5196+1137G>A mutation, whole-genome sequencing (WGS) data for probands as well as unaffected parents were available, enabling phasing of variants in the genomic region harboring *ABCA4*. In total, three probands affected by *ABCA4*-associated retinopathy in our cohort of cases recruited to the Genomics England 100,000 Genomes Project included this allele.

For individual 2, c.5196+1137G>A was identified on the paternal allele *in trans* with the reported missense variant (p.N98S). Filtering of *ABCA4* variants with an allele frequency (AF) of <0.1 in the

Table 1. Clinical Characteristics of *ABCA4*-Associated Retinopathy Patients Harboring c.5196+1137G>A

PID	Institution	Sex	Age at Onset (Years)	Age at Last Examination (Years)	BCVA		Fishmann Classification[a]	Foveal Photoreceptors	Extent of FAF abn. with Regard to Vascular Arcades	Clinical Diagnosis	Age at ERG (Years)	ffERG Group	PERG
					OD	OS							
1	MEH	F	55	60	6/9	6/6	2	spared	beyond	STG	–	–	–
2	MEH	F	22	43	3/60	2/60	4	atrophy	beyond	CRD	41	3	A
3[#]	MEH	M	22	39	3/60	3/60	4	atrophy	beyond	STG	36	1	A
4[#]	MEH	F	23	30	6/24	6/24	3	atrophy	beyond	STG	–	–	–
5	MEH	F	41	64	6/18	CF	4	atrophy	beyond	CRD	61	3	A
6[#]	MEH	M	12	12	–	–	0	early changes	within (minimal)	STG	–	–	–
7	MEH	M	33	44	6/60	6/92	4	atrophy	beyond	STG	42	1	A
8	MEH	F	10	26	6/36	6/36	2	early changes	within (minimal)	STG	21	1	A
9	MEH	F	24	43	6/60	6/60	4	atrophy	beyond	CRD	36	3	A
10[#]	MEH	F	15	21	6/36	6/36	2	atrophy	beyond	STG	15	1	A
11	MEH	M	46	61	6/60	6/60	3	atrophy	beyond	STG	–	–	–
12[#]	MEH	F	22	62	HM	6/60	4	atrophy	beyond	CRD	52	3	A
13	MEH	M	46	56	6/18	6/12	4	spared	beyond	STG	47	1	A
14	MEH	F	25	30	6/6	6/19	2	atrophy	beyond	STG	26	1	N
15	MEH	F	39	51	6/9	6/9	2	early changes	beyond	STG	40	1	A
16[#]	MEH	F	24	50	2/60	6/6	3	atrophy	beyond	STG	39	1	A
17	MEH	F	33	43	3/60	6/12	3	atrophy	beyond	CRD	39	3	A
18	MEH	F	55	62	6/6	6/36	3	spared	beyond	STG	57	1	N
19	MEH	F	10	20	6/48	6/30	2	atrophy	beyond	STG	17	1	A
20	MEH	M	40	72	3/60	3/60	4	atrophy	beyond	STG	68	1	A
21	PAH	F	35	35	6/9	6/9	3	spared	beyond	STG	35	1	N
22a	RUMC	F	13	29	6/60	3/60	3	atrophy	beyond	STG	25	1	A
22b	RUMC	M	27	35	20/60	24/60	2	atrophy	beyond	STG	27	1s	–
23[#]	RUMC	F	4	41	1/60	1/60	4	atrophy	beyond	STG	34	1s	–
24[#]	RUMC	M	15	43	1/60	1/60	4	atrophy	beyond	STG	18	1	–

[a]Fishman classification: I, flecks limited to within the vascular arcades; II, fleck-like lesions anterior to the vascular arcades and/or nasal to the optic disc; III, most diffuse flecks resorbed leaving diffuse RPE atrophy; and IV, not only diffusely resorbed fundus flecks and atrophy of the RPE but also diffuse choriocapillaris atrophy.[1]

[#]Data for individuals who carry c.5196+1137G>A *in trans* with a predicted null allele are indicated with a hash. Patients 22a and 22b are siblings. Individuals 23 and 24 were described previously in Bax et al. (2015).[13] PID, patient ID; BCVA, best corrected visual acuity; OD, right eye; OS, left eye; FAF, fundus autofluorescence; ERG, electroretinography; ffERG, full-field electroretinography; PERG, pattern electroretinography; MEH, Moorfields Eye Hospital, London, UK; F, female; STG, Stargardt disease; –, not available; CRD = cone-rod dystrophy; M, male; PAH, Princess Alexandria Hospital, Brisbane, QLD, Australia; RUMC, Radboud University Medical Center, Nijmegen, the Netherlands; abn.: abnormalities; A: abnormal; N: normal; CF: counting fingers; HM: hand motion; 1s, normal photopic, low scotopic.

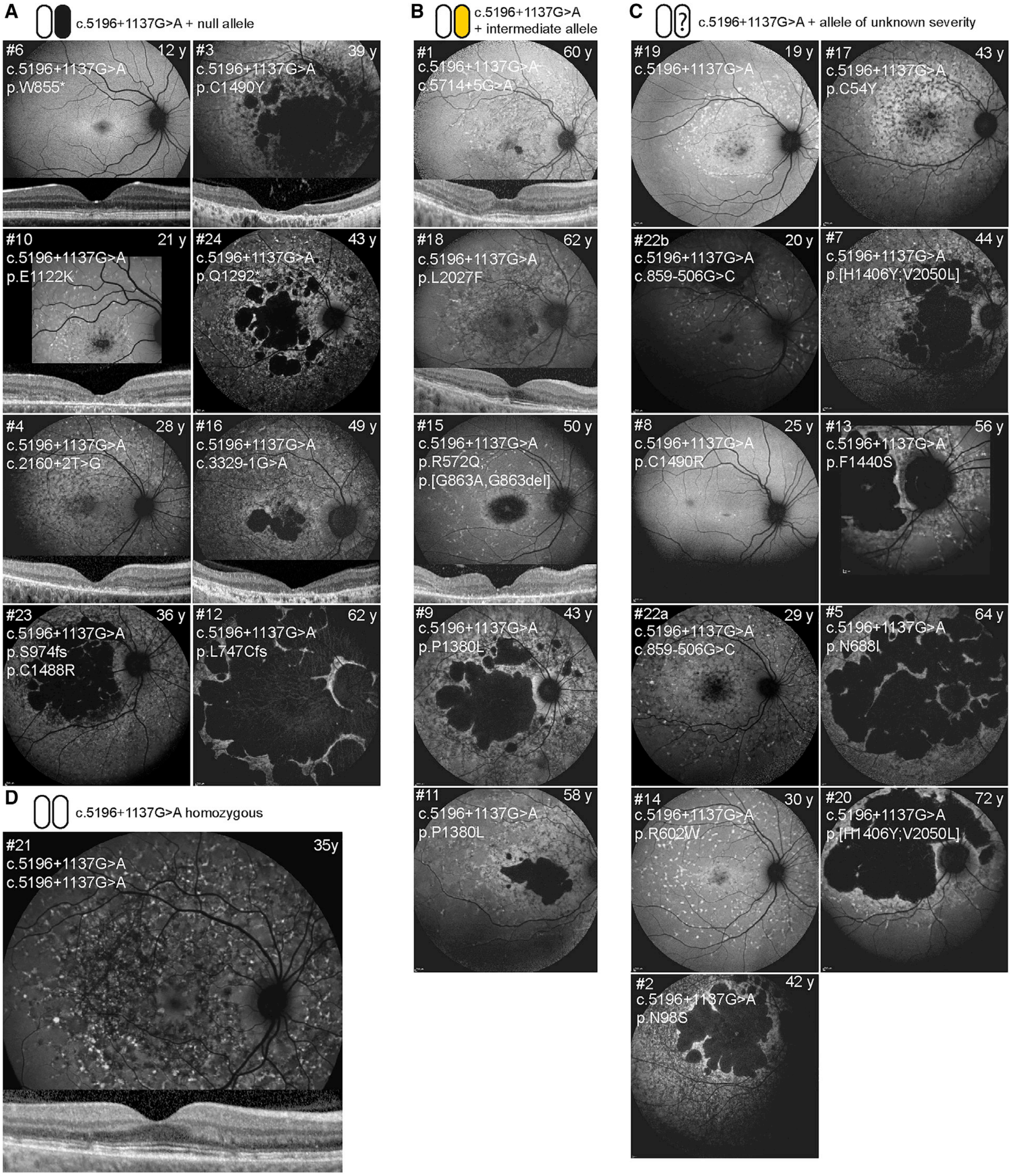

Figure 1. Fundus Autofluorescence Images of *ABCA4*-Associated Retinopathy Cases Harboring c.5196+1137G>A

FAF images of patients harboring c.5196+1137G>A *in trans* with different groups of alleles. Classification of *in trans ABCA4* alleles was performed previously based on electrophysiology.[7] (A) Note the increasing retinal degeneration (no flecks, fleck outside the arcades, absorbed flecks, chorioretinal atrophy) in patients harboring c.5196+1137G>A *in trans* with null, when arranged by age. (B and D) Relatively milder phenotypes can be observed in patients harboring c.5196+1137G>A *in trans* with intermediate alleles c.5714+5G>A, p.L2027F and p.[G863A, G863del] (B) and in the homozygous state (D), even at older ages. (C) Patients harboring c.5196+1137G>A *in trans* with alleles of unknown severity arranged by age exhibiting various FAF patterns.

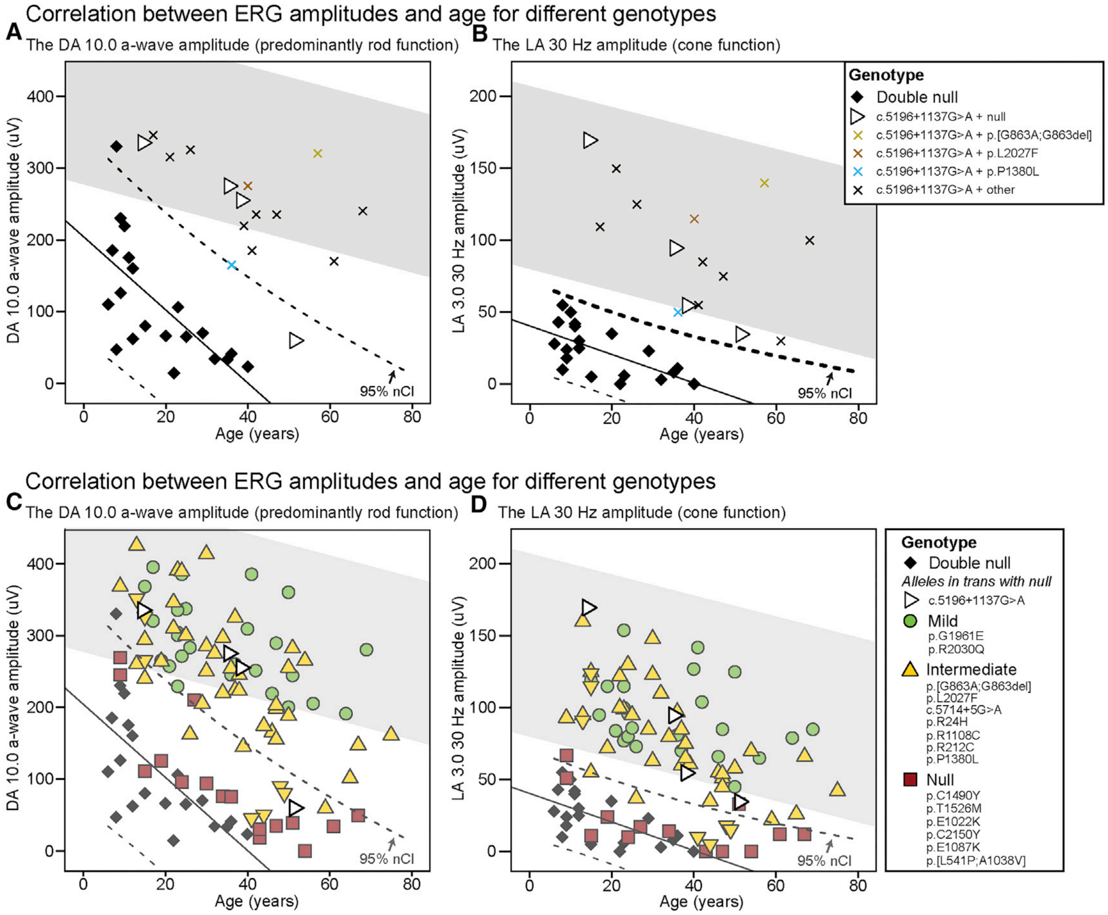

Figure 2. Electroretinography of *ABCA4*-Associated Retinopathy Cases Harboring c.5196+1137G>A

(A and B) The dark-adapted (DA) 10.0 a-wave amplitudes (A) and the light-adapted (LA) 3.0 30-Hz amplitudes (B) of "double null" patients and patients harboring c.5196+1137G>A with different mutations *in trans*, plotted by age. (C and D) The DA 10.0 a-wave amplitudes (C) and the LA 3.0 30-Hz amplitudes (D) of patients harboring c.5196+1137G>A in *trans* with null alleles and patients harboring 15 other alleles of previously determined severity,[7] plotted by age. Data from a single recording performed at the same center (MEH in Table 2) was used for each patient. One patient (ID 17) had non-recordable 30-Hz amplitudes due to noise. The gray area represents the 95% confidence interval (CI) of the healthy volunteers. On each chart, the "double null" patients are indicated as a baseline reference with their regression line and their CIs. Note the significantly better retinal function of patients harboring c.5196+1137G>A in comparison to the "double null" patients.

gnomAD dataset and an in-house cohort of 7,766 alleles from the WGS cohort revealed four variants informative for the haplotype (defined as haplotype B; Table 2; Table S1).

For individual 3, c.5196+1137G>A was identified on the paternal allele *in trans* with the previously reported missense variant (p.C1490Y). Filtering of *ABCA4* variants applying the same criteria revealed a different haplotype (defined here as haplotype C), which contained the variant in intron 36 (Table 2; Table S1).

Individual 1 was recruited for WGS with only an affected sibling; therefore, it was not possible to accurately phase variants located in or near *ABCA4* in the absence of parental samples. However, we did identify the upstream variant chromosome (chr) 1:94,531,618T>C shared with individual 2 (haplotype B) and that was present in only 2 out of 15,430 European alleles in gnomAD in the absence of the downstream variants found on the same haplotype. This may suggest that the haplotypes observed in individuals 1 and 2 have a common ancestral origin that underwent recombination in the *ABCA4* gene region, giving rise to the additional haplotype observed in individual 1 (defined here as haplotype A; Table 2; Table S1). Alternatively, it is possible that the chr1:94,531,618T>C variant was present on the opposite allele, in which case the haplotype would be unknown.

In addition to individuals 1–3, subjects 13 and 14 also underwent WGS as singletons. In both cases, the presence of the four rare variants previously found to define haplotype B were identified. Subsequently, these and other rare variants close to c.5196+1137G>A that marked the different haplotypes (including chr1:94,531,618T>C to determine haplotype A) were genotyped in all available remaining cases via Sanger sequencing (Table S1). An additional 5/17 cases were

suggestive of haplotype A, 3/17 were consistent with haplotype B, and 1/17 were consistent with haplotype C. Of the remaining four cases available for testing, three did not carry any of the haplotype-associated variants, whereas one individual carried all variant alleles tested (Table 2; Table S1). This may be explained by the relatively high frequency of chr1:94,513,853TAA>T in gnomAD (minor AF [MAF] of 0.07479); therefore, it may also be present on the *trans* allele.

Identification of *ABCA4* Variants in Intron 36

Following the clinical characterization of patients harboring the c.5196+1137G>A variant, an in-depth molecular analysis of this and other variants in close proximity (i.e., intron 36 of the *ABCA4* gene) was performed. Previously, a few variants in intron 36 potentially causing *ABCA4*-associated retinopathy have been described, including an *in silico* analysis of the splicing defects they exert.[19] In addition, other reports have described the clustering of deep-intronic variants in certain introns, suggesting that some introns are more prone to harbor disease-causing mutations than others.[10,14] To investigate which *ABCA4* variants in intron 36 could potentially lead to splicing defects (and can be considered pathogenic), all previously described variants were collected and analyzed for frequency statistics and splicing predictions. This led to the identification of 11 additional variants, besides c.5196+1137G>A, that could potentially influence splicing, e.g., by the generation of new splice acceptor or donor sites or changing splicing enhancer or silencer motifs (Table 3; Table S2). Although some of the variants had a relatively high MAF, these were still tested, as previous studies demonstrated a pathogenic role of frequent deep-intronic variants (c.769-784C>T, c.4253+43G>A).[14]

To study the causative nature of all these variants, a new construct was generated that could be used in a midigene splicing assay.[24] This construct, coined BA32, harbors the region of *ABCA4* spanning from intron 34 to intron 38, enabling the study of the splicing of intron 36 in a genomic context more extended than a minigene. Following confirmation that transfection of this midigene, indeed, leads to the expected splicing pattern (merge of exons 35, 36, 37, and 38), site-directed mutagenesis was performed to generate mutant BA32 constructs, each harboring one of the selected deep-intronic variants. Transfection of the wild-type (WT) and the various mutant BA32 constructs into HEK293T cells followed by reverse transcription (RT)-PCR analysis revealed that four out of the 12 variants led to aberrant splicing, i.e., the insertion of additional sequences in between exons 36 and 37 (Figure 3). For two variants—c.5196+1013A>G (M4) and c.5196+1056A>G (M6)—the predominant transcript contained a PE (of 129 or 177 nt, respectively) that was partially identical, which was defined by an already existing cryptic splice acceptor site (SAS) in the reference sequence and the strengthening of different cryptic splice donor sites (SDSs) by either of the variants. Two other variants, c.5196+1137G>A (M9) and c.5196+1216C>T (M11), led to the insertion of another, 73-nt, PE but to different fractions of transcripts. Both variants led to the insertion of the exact same PE, although the c.5196+1137G>A variant slightly strengthens a cryptic SAS, whereas c.5196+1216C>A clearly strengthens a cryptic SDS of the PE (Figures 3A and 3B; Figure S4). Seven out of the other eight variants did not alter the splicing pattern (Figure S5), suggesting that these variants do not have a drastic impact on the processing of *ABCA4* pre-mRNA, at least not in HEK293T cells used here. Variant c.5196+1078del was not assessed, since site-directed mutagenesis to introduce this change into the BA32 construct was repeatedly unsuccessful.

Aberrant Splicing Induced by c.5196+1137G>A Variant in Photoreceptor Precursor Cells

As can be observed in Figure 3B, the splicing defect evoked by the c.5196+1137G>A variant was only present in a small proportion of transcripts following transfection of the mutant midigene into HEK293T cells. Previous reports demonstrated that, for some deep-intronic variants, the degree of PE inclusion can be tissue-dependent or even cell type-dependent; e.g., the splicing defect is more prominent in retina-like tissue.[10,25] Given the association of distinct c.5196+1137G>A haplotypes with patients with *ABCA4*-associated retinopathy, we explored whether this was also true for this variant. Fibroblast cells of affected individual 23 harboring this allele *in trans* with a protein-truncating mutation (p.[S974Qfs*64;C1488R]), together with that of a healthy control individual, were reprogrammed into induced pluripotent stem cells (iPSCs), as described previously.[22] Subsequently, iPSCs were differentiated into photoreceptor precursor cells (PPCs) following a 30-day protocol,[26] giving rise to an increased expression of several retina-specific genes, including *ABCA4*. The degree of differentiation was assessed via qPCR analysis (Figure S6). Prior to harvesting, cells were treated with cycloheximide (CHX), an agent that blocks NMD of aberrant transcripts, as both alleles are predicted to give rise to premature termination codons that can lead to this phenomenon. The band with the correct size could be derived either from the second allele (i.e., NMD is incomplete) or from the correctly spliced product of the allele with the deep-intronic mutation. As there were no SNPs nearby, this could not be further investigated. As shown in Figure 3C, especially following CHX treatment, the degree of PE insertion caused by c.5196+1137G>A was notably higher compared to that in the midigene splicing assay, particularly when taking into account that only half of the *ABCA4* transcripts in these PPCs are derived from the allele harboring this mutation. Of note, a small fraction of transcripts showed PE inclusion in the control PPCs, following CHX treatment, demonstrating that even in the absence of the deep-intronic variant, this PE can be recognized by the splicing machinery in retina-like cells.

AONs Rescue Splicing Defects due to Intron 36 Variants in *ABCA4*

Following the identification of the PE insertions caused by the four variants in intron 36, AONs were designed to block splicing of these PEs into the *ABCA4* mRNA, with the aim of restoring normal splicing. For the partially overlapping PEs (PE129 and PE177) induced by c.5196+1013A>G and c.5196+1056A>G, respectively, four AONs were designed. AON1 and AON2 target a region present in both PEs, whereas AON3 and AON4 overlap with PE177 but not, or at least not entirely, with PE129 (Figure 4A). Of note, AON3 targets a region in PE177 that encompasses the reference nucleotide at position c.5196+1013 and, therefore, contains a mismatch toward

Table 2. Gender and Genotypic Details of STGD1 Cases with c.5196+1137G>A

		Allele 1		Allele 2				
PID	Institution	cDNA	Protein	cDNA	Protein	Segregating	Allele 1 haplotype	Moorfields ID
1	MEH	c.5196+1137G>A	p.[=,M1733Efs*78]	c.5714+5G>A	p.[=,E1863Lfs*33]	ND	A	21567
2	MEH	c.5196+1137G>A	p.[=,M1733Efs*78]	c.293A>G	p.(N98S)	Y	B	4646
3[#]	MEH	c.5196+1137G>A	p.[=,M1733Efs*78]	c.4469G>A	p.(C1490Y)	Y	C	4988
4[#]	MEH	c.5196+1137G>A	p.[=,M1733Efs*78]	c.2160+2T>G	p.(?)	ND	B	20160
5	MEH	c.5196+1137G>A	p.[=,M1733Efs*78]	c.2063A>T	p.(N688I)	ND	B	20122
6[#]	MEH	c.5196+1137G>A	p.[=,M1733Efs*78]	c.2564G>A	p.(W855*)	ND	–	25804
7	MEH	c.5196+1137G>A	p.[=,M1733Efs*78]	c.[4216C>T;6148G>C]	p.[(H1406Y);(V2050L)]	Y	C	21670
8	MEH	c.5196+1137G>A	p.[=,M1733Efs*78]	c.4468T>C	p.(C1490R)	Y	U	20567
9	MEH	c.5196+1137G>A	p.[=,M1733Efs*78]	c.4139C>T	p.(P1380L)	Y	A	19004
10[#]	MEH	c.5196+1137G>A	p.[=,M1733Efs*78]	c.3364G>A	p.(G1122K)	Y	B	20522
11	MEH	c.5196+1137G>A	p.[=,M1733Efs*78]	c.4139C>T	p.(P1380L)	ND	A	4198
12[#]	MEH	c.5196+1137G>A	p.[=,M1733Efs*78]	c.2239del	p.(L747Cfs*40)	Y	A	4617
13	MEH	c.5196+1137G>A	p.[=,M1733Efs*78]	c.4319T>C	p.(F1440S)	Y	A	16063
14	MEH	c.5196+1137G>A	p.[=,M1733Efs*78]	c.1804C>T	p.(R602W)	Y	A	20898
15	MEH	c.5196+1137G>A	p.[=,M1733Efs*78]	c.[1715G>A;2588G>C]	p.[(R572Q;G863A,G863del]	Y	U	20546
16[#]	MEH	c.5196+1137G>A	p.[=,M1733Efs*78]	c.3329-1G>A	p.(?)	Y	U	17478
17	MEH	c.5196+1137G>A	p.[=,M1733Efs*78]	c.161G>A	p.[C54Sfs*14,C54Y]	ND	A	20655
18	MEH	c.5196+1137G>A	p.[=,M1733Efs*78]	c.6079C>T	p.(L2027F)	ND	U	21317
19	MEH	c.5196+1137G>A	p.[=,M1733Efs*78]	unknown	unknown	NA	A	21659
20	MEH	c.5196+1137G>A	p.[=,M1733Efs*78]	c.[4126C>T; 6148G>C]	p.[(H1406Y);(V2050L)]	ND	–	21257
21	PAH	c.5196+1137G>A	p.[=,M1733Efs*78]	c.5196+1137G>A	p.[=,M1733Efs*78]	NA	–	NA
22a	RUMC	c.5196+1137G>A	p.[=,M1733Efs*78]	c.859-506G>C	p.[F287Tfs*32,=]	Y	A	NA
22b	RUMC	c.5196+1137G>A	p.[=,M1733Efs*78]	c.859-506G>C	p.[F287Tfs*32,=]	Y	(A)	NA
23[#]	RUMC	c.5196+1137G>A	p.[=,M1733Efs*78]	c.[2918+775_3328+640del; 4462T>C]	p.[(S974Qfs*64;C1488R)]	Y	A	NA
24[#]	RUMC	c.5196+1137G>A	p.[=,M1733Efs*78]	c.3874C>T	p.(Q1292*)	Y	A	NA

[#]Data for individuals that carry c.5196+1137G>A *in trans* with a predicted null allele are indicated with a hash. Patients 22a and 22b are siblings. For individual 22b, haplotype A was assumed given its relation to individual 22a; hence, the parentheses. PID, patient ID; MEH: Moorfields Eye Hospital, London, UK; ND, not determined; NA, not applicable; –, haplotyping not performed; U, haplotype unknown; PAH: Princess Alexandria Hospital, Brisbane, QLD, Australia; RUMC: Radboud University Medical Center, Nijmegen, the Netherlands;.

Table 3. *ABCA4* Intron 36 Variants and AFs in Population Databases

Intron 36 Variant	cDNA Variant	Genomic Position (hg19)	AF_gnomAD_nFE (Accessed 10-18-2019)	AF_gnomAD (accessed 10-18-2019)	Reference
M1	c.5196+235G>A	94,484,903	–	–	30
M2	c.5196+771G>A	94,484,367	–	–	30
M3	c.5196+899C>T	94,484,239	0.03126	0.02097	15
M4	c.5196+1013A>G	94,484,125	–	–	15
M5	c.5196+1015A>G	94,484,123	0.05542	0.05228	15
M6	c.5196+1056A>G	94,484,082	–	–	30
M7	c.5196+1078del	94,484,060	0.03418	0.05990	15
M8	c.5196+1136C>A	94,484,002	0.0001296	0.01073	11
M9	c.5196+1137G>A	94,484,001	0.0001297	0.00009558	19
M10	c.5196+1159G>A	94,483,979	0.0003888	0.003694	11
M11	c.5196+1216C>A	94,483,922	–	–	19
M12	c.5196+1614G>A	94,483,524	–	–	30

hg19, human genome version 19; AF, allele frequency; nFE, non-Finnish European; –, not detected.

the c.5196+1013A>G change. As shown in Figure 4B, co-transfection of the corresponding midigene with AON1 or AON2 resulted in a full correction of the aberrant splicing events induced by c.5196+1013A>G, AON3 showed only a partial correction, and AON4 had no effect. In contrast, for the c.5196+1056A>G variant, all four AONs showed restoration of correct splicing, with AON1 and AON3 being the most potent (Figure 4C). For the 73-nt PE inclusion arising from either c.5196+1137G>A or c.5196+1216C>A, four AONs were designed, none of which were allele-specific. To assess the efficacy of these AONs to abolish PE inclusion by the c.5196+1216C>A variant, co-transfection of the midigene with the AONs resulted in a complete correction of splicing for AON5 and AON6, whereas AON7 and AON8 showed a partial effect (Figure 4D).

To study splicing defects caused by c.5196+1137G>A, iPSC-derived PPCs were again used, as these show a more robust insertion of PE73 compared to the HEK293T cells transfected with the corresponding mutant midigene. As shown in Figures 5A and 5B, AON6 and AON7 were most potent in converting PE73-containing transcripts to the correct transcript, while AON5 and AON8 had little effect. Of note, the PE-containing transcripts, again, were more visible following CHX treatment. Finally, to study PE73 inclusion upon a longer differentiation period, iPSCs were differentiated for 180 days, i.e., to form 3D retinal organoids. Previously, we have shown that AONs can be used to correct splicing of aberrantly spliced *CEP290* in retinal organoids,[25] and we have used them as part of the preclinical development of an AON-based therapy.[21,27] Retinal organoids were generated from iPSCs derived from individual 15 (Table 2), who carries c.5196+1137G>A *in trans* with a complex allele (p.[R572Q;G863A,G863del;N1868I]), as previously described.[28,29] Retinal organoids developed as shown previously, with maturation of a neuroblastic cell layer into a defined outer-nuclear-layer-like structure, with expression of photoreceptor markers evident by day 120 (data not shown). As retinal organoids matured further to day 180, a "brush border" of outer-segment-like structures appeared at the apical edge of the organoids, which were positive for rhodopsin expression (Figure S7). Organoids were also positive for photoreceptor markers such as recoverin and cone arrestin, and L/M opsins, suggesting the correct formation of the retina-like structure (Figure S7). At this stage, the retinal organoids were treated gymnotically with AON6 or SON for 72 h, in the absence of any NMD inhibition, to mimic the conditions that might be applied *in vivo*. Treatment with AON6 led to a dose-dependent decrease in PE-containing transcripts (Figures 5C and 5D). In all experiments, transfection of a negative control sense oligonucleotide (SON) had no effect on splicing, showing the same results as those in the untreated condition. In addition, all AONs did not seem to have an effect when added to control cells or to those transfected with a WT midigene.

DISCUSSION

In this study, we assessed variants in intron 36 of *ABCA4* and their potential involvement with *ABCA4*-associated retinopathy. Detailed clinical characterization and genomic analysis of the most frequent variant, c.5196+1137G>A, showed that this variant is likely to have arisen on different haplotype backgrounds and acts as an allele of intermediate severity. This is in line with the molecular defects that were found in PPCs and retinal organoids harboring this mutation, which showed a residual amount of a normally spliced product. For 11 other *ABCA4* variants in intron 36, three were also found to result in clear splicing defects, all by inserting PEs into the final *ABCA4* transcript. Furthermore, by using cultured HEK293T cells or patient-derived PPCs and 3D retinal organoids, all splicing defects could be rescued by the administration of AONs targeting these PE inclusions.

The c.5196+1137G>A variant was one of the first deep-intronic mutations described in *ABCA4*.[19] In that study, it was shown that this mutation could lead to splicing that results in the inclusion of a PE in a small proportion of *ABCA4* transcripts in patient-derived

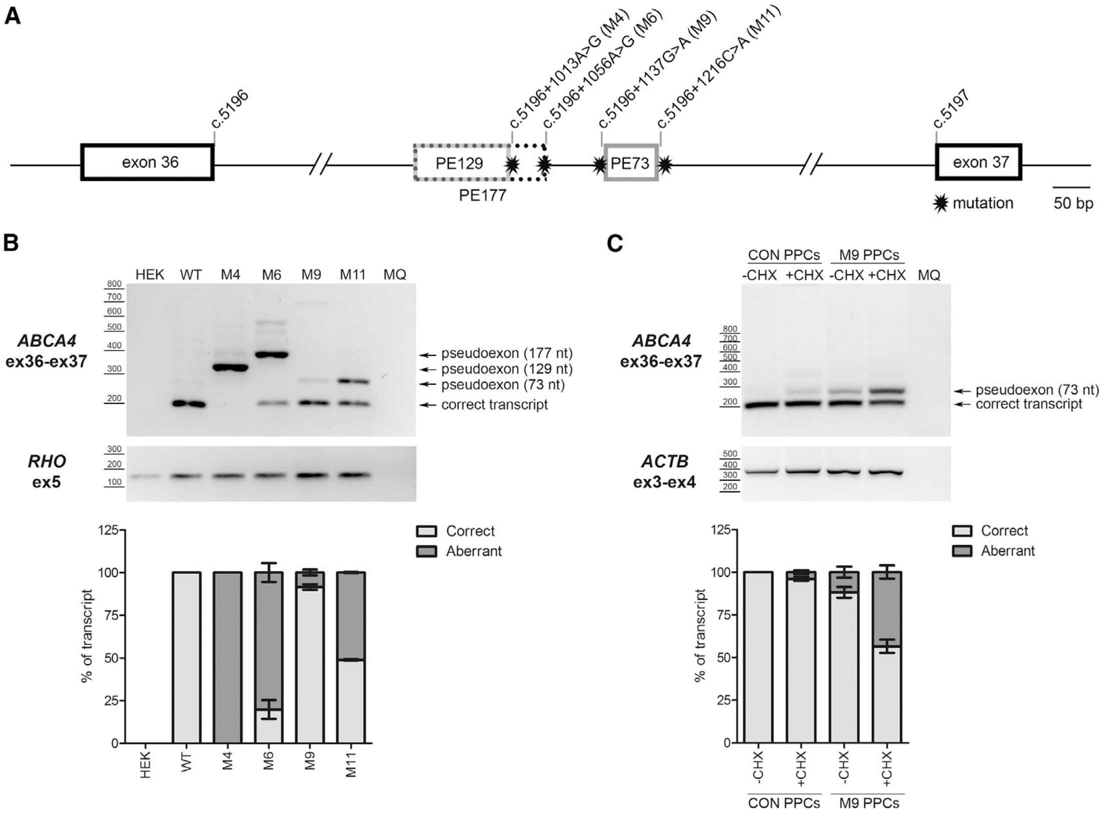

Figure 3. Pseudoexon Insertion Caused by Deep-Intronic Variants in Intron 36

(A) Schematic representation of intron 36 with the location of all the mutations and the pseudoexon (PE) insertion caused by each variant (M). The number of the different PEs indicates the size in nucleotides. (B) Identification of PEs using a midigene-based approach in HEK293T cells. *RHO* amplification was used as transfection and loading control. (C) Identification of a 73-nt PE in patient-derived photoreceptor precursor cells (PPCs). This PE is subjected to NMD, as its detection is clearly increased upon cycloheximide (CHX) treatment. *ACTB* was used as a loading control. MQ, the negative control of the PCR. Semiquantitative analysis of the ratio of aberrant and correct transcripts is indicated in the graphs below each electrophoresis gel. In all graphs, results are presented as average ± SD.

keratinocytes. These data are similar to the observations made here, where the PE insertion was at a low level in our midigene splicing assay that was performed in HEK293T cells. Only when differentiating patient-derived cells to a more retinal lineage, either as PPCs or retinal organoids, did the proportion of PE-containing *ACBA4* transcripts increase, as has been described for other splicing defects due to deep-intronic changes.[10,25] Despite a relatively high frequency of this allele in patients, there has remained some controversy about the pathogenic nature of the c.5196+1137G>A variant, based on the AF of this variant in African American control subjects in a single study but mainly on the notion that, in macaques, c.5196+1137A (the mutant allele in humans) was found to be the most frequent major allele at this position. In macaque retina harboring this A allele, no PE insertion was detected; thus, the pathogenicity of this variant was questioned.[30] However, when directly comparing the sequence of the entire region encompassing the PE in humans, several additional nucleotide differences were identified between the human and macaque genomic sequence either in the 73-nt PE or in the cryptic splice donor site (Figure S8). Whereas Human Splicing Finder (HSF) software attributes equal scores to the human mutant and macaque splice acceptor and donor sites, another splicing prediction software, MaxEntScan, predicts a significantly lower strength of the SDS in macaque compared to human. In addition, a number of the differences between human and macaque within the PE are predicted to generate splicing silencer sequences in macaque but not in human. Since pre-mRNA splicing is well known to be a complex process involving not only the splice acceptor and donor site sequences but also the binding sites for exonic splicing enhancers and silencers,[31] this divergence may explain why c.5196+1137G>A induces PE insertion in humans but not in macaques.

Patients carrying the c.5196+1137G>A allele *in trans* with known disease alleles had clinical presentations within the spectrum of *ABCA4* retinopathy, supporting the pathogenicity of the allele. In addition, using WGS, no more plausible potentially pathogenic variant was identified in the patients that could exclude the pathogenicity of c.5196+1137G>A. Although the majority of cases exhibited FAF abnormalities beyond the vascular arcades, and the fovea was rarely spared, the retina-wide photoreceptor dysfunction was relatively mild, as demonstrated by preserved peripheral cone and rod function on the ffERG in the majority of subjects. Categorization of the

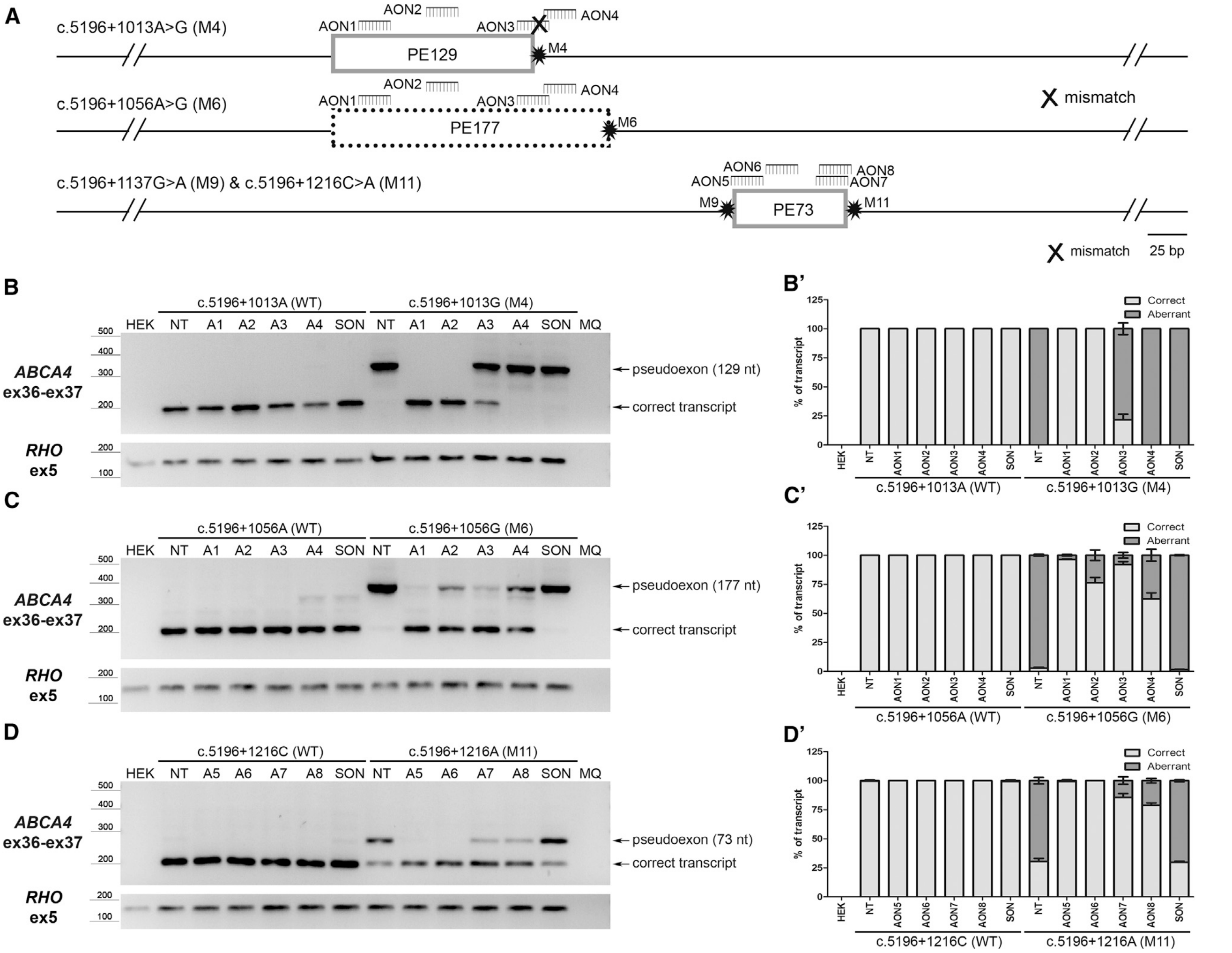

Figure 4. Assessment of Splicing Redirection by AONs Using a Midigene-Based System in HEK293T Cells

(A) Schematic representation of the pseudoexons (PEs) introduced by each variant (M) and the relative position of the different AONs within the region. (B and B') Assessment by RT-PCR of AON1 to AON4 (A1 to A4) in HEK293T cells transfected with either the WT midigene or the midigene containing the c.5196+1013A>G (M4) variant (B) and its corresponding semi-quantification expressed in the ratio of correct to aberrant transcript (B'). (C and C') Splicing redirection by AON1 to AON4 for the c.5196+1056A>G (M6) variant in HEK293T cells (C) and its semi-quantification expressed in the ratio of correct to aberrant transcript (C'). (D and D') Effect of AON5 to AON8 (A5 to A8) in redirecting the splicing defect introduced by c.5196+1216C>A (M11) in HEK293T cells (D) and its semi-quantification expressed in the ratio of correct to aberrant transcript (D'). In all cases, a scrambled oligonucleotide (SON) was used as negative control. MQ, the negative control of the PCR. In all graphs, results are presented as average ± SD.

c.5196+1137G>A allele was performed by selecting a subgroup of patients harboring this allele *in trans* with a (predicted) null allele and comparing their ages at onset, ERG amplitudes, and FAF patterns with those of patients harboring two null alleles or one of the 15 *ABCA4* alleles of known severity,[7] *in trans* with null alleles. The c.5196+1137G>A was similar to alleles of intermediate severity. These had been defined previously as alleles that, *in trans* with null, produce a significantly milder phenotype than two null alleles; however, these often progress to cone-rod dystrophy (CRD) at older ages. The intermediate severity of this variant is in line with other findings, i.e., none of the cases harboring c.5196+1137G>A carried a mild allele *in trans* (e.g., p.G1961E and p.R2030Q, which, *in trans* with null, produce normal ffERG even at older ages),[7] as it is likely that such a combination would result in ABCA4 functional protein reduction not surpassing the disease threshold. In addition, a recent study on deep-intronic *ABCA4* variants in a French cohort also concluded that the c.5196+1137G>A variant was mostly associated with a milder phenotype, based on six subjects harboring this allele.[32] It should also be noted, however, that evidence is emerging that some variants that were long considered pathogenic or benign by themselves, such as c.2588G>C; p.[G863A,G863del] and c.5603A>T; p.(N1868I), are fully penetrant only when in combination on the same allele. The same is true for the *cis* configuration of p.(N1868I) with a recently identified intronic mutation, c.769-784C>T.[9,33,34] In addition, modifier

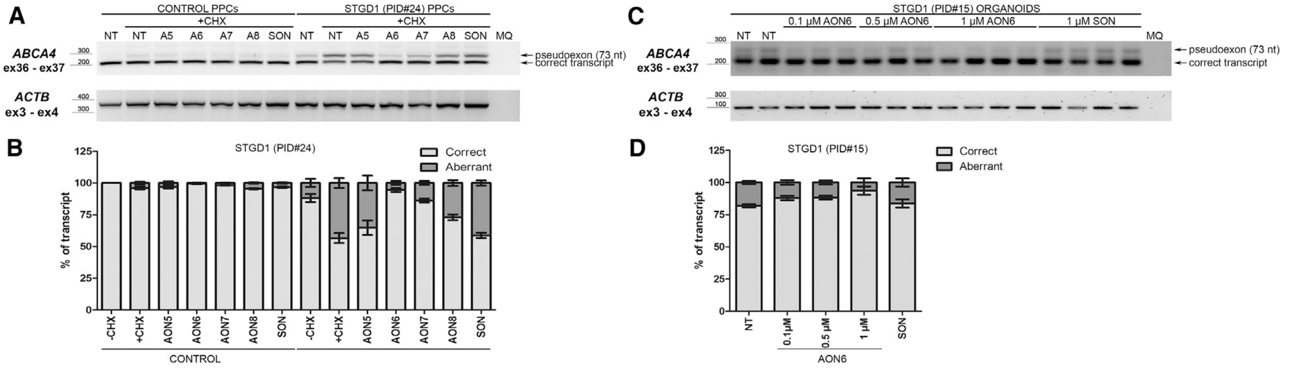

Figure 5. AON-Based Splicing Correction for the c.5196+1137G>A, M9, Variant Using Patient-Derived Retina-like Cells

(A) RT-PCR analysis of control and STGD1 individual PPCs treated with AON5 to AON8 (A5 to A8) or scrambled oligonucleotide (SON). Pseudoexon detection was enhanced by cycloheximide (+CHX) treatment. *ACTB* amplification was used as loading control. (B) Representation of the ratio of correct to aberrant transcript upon semi-quantification. (C) RT-PCR analysis of STGD1 individual-derived, 185-day-old retinal organoids treated with AON6 at the indicated concentrations or SON. *ACTB* amplification was used as loading control (of note, primers were different from those used in B). (D) Graphical representation of the ratio of correct to aberrant transcript upon semi-quantification. MQ, the negative control of the PCR. In all graphs, results are presented as average ± SD.

variants, either within or outside of *ABCA4*, as well as environmental factors will likely play a role in determining disease outcome.[9] Thus, care is warranted when attributing a severity score to individual alleles. The existence of residual ABCA4 function produced by the c.5196+1137G>A variant is also supported by the clinical presentation of the homozygous patient; namely, a significantly delayed disease onset and better preserved photoreceptors in comparison to the patients with only a single c.5196+1137G>A allele in combination with a null allele. Finally, the molecular studies revealed that the PE insertion induced by this variant was only present in a proportion of the *ABCA4* transcripts, thus always giving rise to residual WT transcript and translation of a functional protein.

The fact that the c.5196+1137G>A variant was identified on different haplotypes suggests that it may have arisen independently, which would further support a case for pathogenicity. However, an alternative explanation is that the C haplotype is ancestral and that haplotype A occurred by a centromeric recombination, followed by a telomeric recombination giving rise to haplotype B. Of note, no clear correlation between the severity of the clinical phenotype and the nature of the haplotypes (A, B, or C) was observed. This suggests that the surrounding variant landscape is unlikely to play an important role in the degree of pathogenicity, although the numbers of cases were small.

In this study, we selected 12 *ABCA4* variants in intron 36 that were predicted to affect pre-mRNA splicing. Upon testing in our midigene assay, however, only three of them showed clear splicing aberration in HEK293T cells. Given the high prevalence of the c.5196+1137G>A change, this variant was further analyzed in retina-like cells to identify potential "retina-specific" splicing defects as previously reported for other variants in *ABCA4* and *CEP290*.[10,25] For the other eight, a similar tissue-specific mechanism may be operational so that it is not possible to exclude pathogenicity solely based on the midigene assay in HEK293T. Thus, other *in vitro* systems alternative to the costly and time-consuming retinal progenitor cell/organoid assays are needed to assess the pathogenic nature of such variants in a high-throughput manner.

For each of the two overlapping PEs that we identified, we designed four AONs to redirect the aberrant splicing processes. All AONs were ordered with a 2′-O-methyl sugar modification and a phosphorothioate backbone (commonly called 2OMe/PS AONs), since another recently developed and widely adopted chemistry (2′-O-methoxyethyl) was not commercially available at the time of the first experiments. Changing chemistries may affect the efficacy of certain AONs and, thus, can be considered for future experiments. Similar to our previous findings,[10,14,35] not all AONs were effective. In general, AONs targeting certain predicted splicing enhancer motifs (SC35), a melting temperature above 48°C and having a GC content between 40% and 60% are good predictors of efficacy,[35,36] yet for every PE this needs to be empirically determined. Given the low number of AON molecules tested, no detailed correlation between the parameters of the selected AONs and their efficacy was possible. As we observed previously,[10,35] a single mismatch between an AON and its target sequence was sufficient to completely abrogate its splicing modulation capacity. AON3, which was designed to block the 129-nt and 177-nt PE inclusion caused by the c.5196+1013A>G and c.5196+1056A>G variant, respectively, harbored the c.5196+1013G allele in its sequence and was, indeed, only effective for the c.5196+1013A>G variant. For the two variants that create the same PE, c.5196+1137G>A and c.5196+1216C>A, the efficacy of the AONs was tested in two different systems. Interestingly, the results were similar but not entirely matching. When AON delivery was combined with the midigene assay in HEK293T cells for the c.5196+1216C>A variant, AON5 and AON6 were the two most efficacious ones, followed by a partial effect of AON7 and AON8. In contrast, while AON6 also remained the most efficacious in the iPSC-derived PPCs harboring the c.5196+1137G>A variant, the ability of AON5 to redirect splicing was hardly detectable. This discrepancy is most likely due to the limitations in the test systems; e.g., the delivery to HEK293T

cells is easier than that to iPSC-derived PPCs. In addition, the molecular context is different: iPSC-derived PPCs have the entire 128-kb *ABCA4* pre-mRNA expressed, while in the midigene assay, only an artificial fragment of 7.5 kb is expressed. In all cases, the effective AONs were able to redirect splicing instead of degrading the transcript, as there was also an increase of correctly spliced transcript.

RNA therapies are gaining momentum for the treatment of a variety of diseases, both inherited and acquired ones.[37] For inherited retinal diseases, the first clinical trial based on AONs to redirect a splicing defect in *CEP290* has recently shown promising results,[21] whereas a second one, for *USH2A*, is currently ongoing (ClinicalTrials.gov: NCT03780257). We have used the same retinal organoid technology that was used in the proof-of-concept studies for the preclinical development of the *CEP290* therapy[27] to show that our AON approach for c.5196+1137G>A is effective, which might facilitate a faster translation to the clinic. Both of the ongoing clinical trials consist of unilateral intravitreal delivery of AONs to patients harboring the corresponding mutation in either a homozygous or compound heterozygous state. AONs are administered every 6 months, since these AONs have been chemically modified to enhance cellular uptake and stability. However, further improvement of the stability and/or delivery of these molecules would reduce the burden for the patients—as well as health care costs—for this type of therapy even more. Alternatives include a viral delivery (e.g., AONs packaged into adeno-associated viruses),[38,39] although these strategies also come with downsides.

In conclusion, in this study, we demonstrate a cluster of *ABCA4* variants in intron 36 which, all in their own way, result in aberrant processing of *ABCA4* pre-mRNA. While some variants appear to act in a cell-independent manner, a recurrent deep-intronic variant in *ABCA4* (c.5196+1137G>A) shows a more retina-specific defect. Detailed clinical characterization of patients harboring this variant show that the severity of the phenotype largely depends on the nature of the second *ABCA4* allele *in trans*, suggesting that the c.5196+1137G>A allele in itself can be considered as an allele of intermediate severity. Finally, we show in patient-derived PPCs and 3D retinal organoids that AONs appear to be a promising tool to correct splicing defects associated with the pathogenic variants identified in this study, warranting further development of these molecules toward clinical trials in order to halt the progression of this disease.

MATERIALS AND METHODS

Subjects

Twenty-five individuals with *ABCA4*-associated retinopathy, all carrying a recurrent variant in intron 36 of *ABCA4* (GRCh37 [hg19]:1:94,484,001C>T, NM_000350.3: c.5196+1137G>A) were recruited for this study. Twenty patients were ascertained from the genetic records of the Inherited Eye Disease clinics at Moorfields Eye Hospital London, UK, while four (two of which were siblings) were ascertained from the Radboud University Medical Center in Nijmegen, the Netherlands. One individual was identified and recruited from a cohort at the Princess Alexandra Hospital in Brisbane, QLD, Australia. For all British and Australian individuals, *ABCA4* mutation screening was performed by next-generation sequencing. Specifically, five individuals underwent WGS analysis, either as part of the Genomics England 100,000 Genomes Project (patient IDs [PIDs] 1–3) or as part of the NIHR-RD study (PIDs 13 and 14).[40] Individuals 4–12 and 15–21 were sequenced as part of the retinal panel at the Molecular Vision Lab (https://www.molecularvisionlab.com), as described elsewhere.[7] For Dutch individuals 22 and 23, haloplex-based *ABCA4* targeted sequencing was performed,[14] while for individual 24, array-based *ABCA4* sequencing was done.[41] Genotypic data are listed in Table 2. Written informed consent adhering to the Declaration of Helsinki was obtained from all individuals prior to participation in the study. This study was approved by the Institutional Review Board (IRB)/Ethics Committee (12/LO/0141/AM2) in the UK and the Medical Ethical Committee 2010-359 (Protocol no. 2009-32; NL no. 34152.078.10) and the Commissie Mensgebonden Onderzoek Arnhem-Nijmegen (dossier no. 2015-1543; dossier code sRP4h) in the Netherlands.

Clinical Analysis

All individuals underwent complete clinical examination by an experienced ophthalmologist and were diagnosed with STGD1 or CRD. Collected phenotype data included age at disease onset, visual acuity, FAF, OCT, ffERG, and pattern ERG (PERG). Disease onset was defined as the age when patients first noticed visual problems. ERG incorporated the recommendations of the International Society for Clinical Electrophysiology of Vision.[42] PERG was used to study the function of the macula, and ffERG was used to study the function of the peripheral retina. FAF and OCT (Spectralis, Heidelberg, Germany) were used to determine the structural integrity of photoreceptors and RPE. Each of the persons exhibited at least one of the following clinical features of STGD1: characteristic yellow-white pisciform flecks in the RPE of the posterior pole that were hyperautofluorescent on FAF imaging or progressive atrophy of the macular RPE.[14] For each individual, clinical details are presented in Table 1.

Patients were classified into three groups according to the ffERG results, as described previously (1 = normal, 2 = cone dystrophy, and 3 = CRD).[23] The dark-adapted (DA) 10.0 a-wave amplitudes (representing mostly rod function) and light-adapted (LA) 30-Hz amplitudes were plotted on a chart to inspect correlation with age and genotype. Only the amplitudes of patients recorded at the same institution (MEH; n = 16) were included for this analysis due to different equipment and recording environment of other institutions, which may affect amplitudes. Since there was a high similarity of ffERG responses from both eyes, the right eye was chosen for the analysis.

To determine the severity of the c.5196+1137G>A allele, a subgroup of patients harboring this variant *in trans* with an allele expected to produce no or minimal ABCA4 function was analyzed (n = 8; indicated in bold in Table 2). This enabled us to isolate the effect of the c.5196+1137G>A allele on the severity of the phenotype. Comparative groups consisted of 102 patients harboring different combinations of *ABCA4* alleles with previously determined severity.[7] The

patient that was homozygous for the c.5196+1137G>A variant was compared to one carrying this allele *in trans* with a null allele.

Haplotype Analysis

Where additional family-member WGS data were available (individuals 1–3), phasing of informative variants to identify those *in cis* with c.5196+1137G>A was possible, thereby establishing the haplotypes on which this mutation was present in those individuals. Single-nucleotide variants with MAF < 0.1 in the gnomAD dataset were identified in the *ABCA4* gene region. Variants present in the carrier parent, and absent in the other parent, were assumed to be *in cis* with the c.5196+1137G>A variant. Once these haplotypes and informative variants were established, direct variant interrogation in the singleton (unphased) WGS data (National Institute for Health Research BioResource – Rare Diseases [NIHR-RD], individuals 13 and 14) was performed to identify which (if any) haplotype was most plausible in these individuals. Direct Sanger sequencing of selected informative variants was performed in the remaining individuals. Primer sequences are provided in Table S3.

Selection of *ABCA4* Intron 36 Variants

To select additional deep-intronic variants in intron 36 of *ABCA4*, a literature search was conducted. All variants in intron 36 of *ABCA4* that were reported in affected individuals were selected based on at least two of the three following selection criteria: (1) variants have an AF of ≨0.005 in general population databases such as in the non-Finnish European (nFE) gnomAD (https://gnomad.broadinstitute.org/) or Genome of the Netherlands (GoNL; http://www.nlgenome.nl); (2) variants cause an alteration in the SAS or SDS scores; and (3) there is a change in predicted splice enhancer or splice silencer elements when compared to the reference sequence. Reference and mutant sequences were analyzed using five algorithms (SpliceSiteFinder-like, MaxEntScan, NNSPLICE, GeneSplicer, and Human Splicing Finder),[43–47] via Alamut Visual software v.2.7. By applying these criteria, 12 variants in intron 36 of *ABCA4* were selected. Details of the selected variants and the *in silico* analyses are shown in Tables 3 and S2.

Cell Lines and Culture Conditions

Human embryonic kidney 293T (HEK293T) cells were purchased from European Collection of Authenticated Cell Cultures (ECACC) (catalogue no. 12022001; Salisbury, UK), and genomic integrity was tested by Short tandem repeats (STR) PCR. HEK293T cells were cultured and passaged twice a week as described previously.[14]

Midigene-Based *In Vitro* Splicing Assay

The individual effect of c.5196+1137G>A and 11 additional selected deep-intronic variants was assessed by midigene-based splicing assays using a newly designed WT construct (coined BA32) harboring a region of *ABCA4* spanning from intron 34 to intron 38. The reference sequence was derived from the bacterial artificial chromosome clone CH17-325O16 (insert g.94,434,639–94,670,492), as described previously.[24] Details of primers used for Gateway cloning, PCR, and/or sequencing are provided in Table S3. The BA32 construct was used as a template to generate mutant midigene constructs by site-directed mutagenesis, which were subsequently validated by Sanger sequencing. Details of mutagenesis and sequencing primers are given in Table S3. WT and mutant BA32 constructs were transfected in HEK293T cells, and the extracted total RNA was subjected to RT-PCR, as previously described.[24] Rhodopsin (exon 5) amplification was used as transfection and loading control. All experiments were performed in two independent replicates. Primer sequences used for RT-PCR analysis are presented in Table S3.

AONs

AON molecules were designed as described previously.[48,49] Briefly, the pseudoexon region, including 50 bp up- and downstream, was subjected to RNA structure and splicing enhancer factor analyses using several freely available software programs. AONs were ordered from Eurogentec with a 2′ O-methyl sugar modification and a phosphorothioate backbone. A SON with the same chemical modifications was used as a negative control. All AON sequences and characteristics are provided in Table S4.

In Vitro Rescue Studies in HEK293T Cells Using Midigenes and AONs

WT and mutant midigene constructs were transfected in HEK293T cells as described earlier. Subsequently, cells were transfected with AONs targeting the PE of which the insertion was induced by the different mutations. AONs were delivered as described previously.[14] Forty-eight h post-AON treatment, cells were harvested, and total RNA was extracted, which was then subjected to RT-PCR. Rhodopsin amplification was used as transfection and loading control. All experiments were performed in two independent replicates. Primer sequences can be found in Table S3.

Rescue Studies in Patient-Derived PPCs

Patient-derived fibroblast cells were reprogrammed to iPSCs using the Yamanaka factors,[50] together with a control line derived from a healthy individual. The pluripotency of these cells was validated by qPCR and immunocytochemistry for pluripotency markers, as described previously.[10] Subsequently, cells were subjected to a two-dimensional differentiation protocol to allow the formation of retinal precursor cells, as described elsewhere.[26] For this, iPSCs were dissociated and seeded in Matrigel-coated 12-well plates and differentiated for 30 days. On day 28, AONs were added to the cells at a final concentration of 1 μM. The day after, cells were subjected to CHX (final concentration, 100 μg/mL) treatment to block NMD. On day 30, cells were harvested and subjected to RNA analysis. RNA was isolated using the Nucleospin RNA Kit (Macherey-Nagel, Düren, Germany) following the manufacturer's instructions. One microgram of RNA was converted into cDNA using SuperScript VILO Master Mix (Invitrogen, Carlsbad, CA USA). Fifty nanograms of cDNA were used for PCR analysis, as described in previous sections. Primers used for validation of the differentiation into PPCs are provided in Table S3.

Quantification of RT-PCR Products

To assess the quantity of the aberrantly and correctly spliced RT-PCR products, densitometric analysis of the gel electrophoresis images was

performed with ImageJ software,[51] after which the ratio of aberrantly spliced products was calculated.

Rescue Studies in Patient-Derived Retinal Organoids

Patient fibroblasts were reprogrammed as previously described.[52] iPSCs were maintained in Essential 8 Flex media (Thermo Fisher Scientific), and retinal organoids were produced as described previously, with modifications.[25,28] Briefly, iPSCs were dissociated into a single-cell suspension and resuspended at 9,000 cells per well of V-bottomed 96-well plates, in E8Flex media supplemented with 20 μM Y-27632 (Millipore) from day 0 and, from day 2, EB2 media supplemented with 20 μM Y-27632, 3 mM IWR1e (Calbiochem), and 1% Matrigel. Media were changed every 2 days. On day 6 only, media were additionally supplemented with 55 ng/mL BMP4 (PeproTech). From day 12 onward, EB2 media were supplemented with 10% fetal bovine serum (FBS) and 100 nM SAG (Tocris). On day 20, embryoid bodies were transferred to U-bottomed 96-well plates, and media were changed to NR media (DMEM/F12 with 10% FBS, 1× N2 supplement [Thermo Fisher Scientific], and 0.5 μM retinoic acid [RA]), replenished every 2 days. Around this time point, retinal organoid formation could be visualized. On day 40, embryoid bodies with clearly defined neuroepithelial structure suggestive of retinal organoids (∼30%) were transferred to 25-well square Petri dishes, and media were changed twice a week. At day 100, RA was withdrawn to encourage formation of outer segments. At day 180 (when the "brush border" was visible at the apical edge), retinal organoids were treated gymnotically with 0.1 μM, 0.5 μM, or 1 μM AON6 or 1 μM SON for 72 h, before processing for analysis.

For RNA analysis, organoids were washed gently with PBS twice before RNA extraction via the RNeasy Plus Micro Kit, as per manufacturer's instructions (QIAGEN). cDNA was transcribed using oligo(dT) primer via the Tetro cDNA Synthesis Kit (Bioline). PCR was performed using GoTaq Green with standard cycling conditions (Promega). Primers are listed in Table S3. Gel band densitometry analysis was performed in ImageJ by measuring average pixel density. For immunofluorescence, organoids were washed gently with PBS once before fixation in 4% paraformaldehyde (PFA)/5% sucrose for 45 min at 4°C. Organoids were cryoprotected using increasing concentrations of sucrose (6.25% and 12.5%) for 30 min each at 4°C before overnight cryoprotection in 25% sucrose at 4°C. Organoids were frozen in OCT Embedding Matrix using cooled acetone before cryosectioning at 10 μm. Sections were processed for immunofluorescence as previously described.[25] Primary antibodies were anti-mouse rhodopsin 4D2 (1:1,000; Merck Millipore), anti-mouse cone arrestin clone 7G6 (1:100; a gift from Peter MacLeish), anti-rabbit L/M opsin (1:500; Merck Millipore), and anti-rabbit recoverin (1:500; Merck Millipore). Secondary antibodies were donkey anti-mouse Alexa Fluor 488 and donkey anti-rabbit Alexa Fluor 555 (1:1,000; Thermo Fisher Scientific).

Statistical Analysis

Statistical analysis was performed using SPSS software v.22 (IBM SPSS Statistics, IBM, Chicago, IL, USA). The Mann-Whitney U test was used to test for significant differences in the median age of onset between patients with different genotypes. The 95% confidence interval of the double null regression line was used to determine whether the ERG amplitudes of patients harboring c.5196+1137G>A differed significantly from those harboring two null alleles.

SUPPLEMENTAL INFORMATION

Supplemental Information can be found online at https://doi.org/10.1016/j.omtn.2020.06.007.

AUTHOR CONTRIBUTIONS

M.K., G.A., A.F., F.P.M.C., M.E.C., A.G., and R.W.J.C. designed the study. M.K., G.A., A.F., D.A.P., P.P.A.D., S.A., N.M.B., L.D., M.N., K.L.H., E.B., E.R.S., and D.P. performed research. M.C.H., C.B.H., and A.R.W. provided clinical data. C.B.H., A.R.W., F.P.M.C., M.E.C., A.G., and R.W.J.C. supervised research. M.K., G.A., A.F., A.G., and R.W.J.C. wrote the first draft of the manuscript. M.K., G.A., A.F., M.E.C., A.G., and R.W.J.C. completed the manuscript. All authors read and approved the content of the manuscript.

CONFLICTS OF INTEREST

R.W.J.C., A.G., F.P.M.C., and M.E.C. are inventors on a filed patent application that is related to the contents of this manuscript. All other co-authors declare no competing interests.

ACKNOWLEDGMENTS

First, we thank the patients and families who kindly participated in this study. We acknowledge the technical assistance of Jan Amsu, Eline Manders, Saskia van der Velde-Visser en Marlie Jacobs-Camps, and the clinical assistance of Anthony G. Robson. This work was supported by the Algemene Nederlandse Vereniging ter Voorkoming van Blindheid, Stichting Blinden-Penning, Landelijke Stichting voor Blinden en Slechtzienden, Stichting Oogfonds Nederland, Stichting Macula Degeneratie Fonds, and Stichting Retina Nederland Fonds (who contributed through UitZicht 2015-31 and 2018-21), together with the Rotterdamse Stichting Blindenbelangen, Stichting Blindenhulp, Stichting tot Verbetering van het Lot der Blinden, Stichting voor Ooglijders, and Stichting Dowilvo (to A.G. and R.W.J.C.). This work was also supported by the Foundation Fighting Blindness USA (grant no. PPA-0517-0717-RAD to S.A., F.P.M.C., A.G., and R.W.J.C.), the Retina UK (grant no. GR596 to F.P.M.C. and R.W.J.C), and the European Union, Marie Curie Sklodowska Action Initial Training Network StarT (grant agreement 813490 to F.P.M.C., M.E.C., and R.W.J.C). In addition, this work was supported by the National Institute for Health Research (NIHR) Biomedical Research Centre at Moorfields Eye Hospital and the UCL Institute of Ophthalmology, the NIHR BioResource – Rare Diseases, a Fight For Sight UK Early Career Investigator Award (to G.A.), the NIHR Great Ormond Street Hospital Biomedical Research Centre at Great Ormond Street Institute of Child Health and Fight for Sight UK (to A.R.W. and M.E.C.), The Wellcome Trust (to M.E.C.), and Moorfields Eye Charity. Finally, this work was supported by a grant from the NIHR Bioresource Translational Research Consortium for Rare Disease (to A.R.W. and A.F.). This research was made possible through access

to the data and findings generated by the 100,000 Genomes Project. The 100,000 Genomes Project is managed by Genomics England (a wholly owned company of the Department of Health and Social Care). The 100,000 Genomes Project is funded by the National Institute for Health Research and NHS England. The Wellcome Trust, Cancer Research UK, and the Medical Research Council have also funded research infrastructure. The 100,000 Genomes Project uses data provided by patients and collected by the National Health Service as part of their care and support. None of the funding organizations had a role in the design or conduct of this research.

REFERENCES

1. Tanna, P., Strauss, R.W., Fujinami, K., and Michaelides, M. (2017). Stargardt disease: clinical features, molecular genetics, animal models and therapeutic options. Br. J. Ophthalmol. *101*, 25–30.

2. Allikmets, R., Singh, N., Sun, H., Shroyer, N.F., Hutchinson, A., Chidambaram, A., Gerrard, B., Baird, L., Stauffer, D., Peiffer, A., et al. (1997). A photoreceptor cell-specific ATP-binding transporter gene (ABCR) is mutated in recessive Stargardt macular dystrophy. Nat. Genet. *15*, 236–246.

3. Quazi, F., Lenevich, S., and Molday, R.S. (2012). ABCA4 is an N-retinylidene-phosphatidylethanolamine and phosphatidylethanolamine importer. Nat. Commun. *3*, 925.

4. Conley, S.M., Cai, X., Makkia, R., Wu, Y., Sparrow, J.R., and Naash, M.I. (2012). Increased cone sensitivity to ABCA4 deficiency provides insight into macular vision loss in Stargardt's dystrophy. Biochim. Biophys. Acta *1822*, 1169–1179.

5. Cornelis, S.S., Bax, N.M., Zernant, J., Allikmets, R., Fritsche, L.G., den Dunnen, J.T., Ajmal, M., Hoyng, C.B., and Cremers, F.P. (2017). In Silico Functional Meta-Analysis of 5,962 ABCA4 Variants in 3,928 Retinal Dystrophy Cases. Hum. Mutat. *38*, 400–408.

6. Cremers, F.P.M., van de Pol, D.J., van Driel, M., den Hollander, A.I., van Haren, F.J., Knoers, N.V., Tijmes, N., Bergen, A.A., Rohrschneider, K., Blankenagel, A., et al. (1998). Autosomal recessive retinitis pigmentosa and cone-rod dystrophy caused by splice site mutations in the Stargardt's disease gene ABCR. Hum. Mol. Genet. *7*, 355–362.

7. Fakin, A., Robson, A.G., Chiang, J.P., Fujinami, K., Moore, A.T., Michaelides, M., Holder, G.E., and Webster, A.R. (2016). The Effect on Retinal Structure and Function of 15 Specific ABCA4 Mutations: A Detailed Examination of 82 Hemizygous Patients. Invest. Ophthalmol. Vis. Sci. *57*, 5963–5973.

8. Fakin, A., Robson, A.G., Fujinami, K., Moore, A.T., Michaelides, M., Pei-Wen Chiang, J., E Holder, G., and Webster, A.R. (2016). Phenotype and Progression of Retinal Degeneration Associated With Nullizigosity of ABCA4. Invest. Ophthalmol. Vis. Sci. *57*, 4668–4678.

9. Runhart, E.H., Sangermano, R., Cornelis, S.S., Verheij, J.B.G.M., Plomp, A.S., Boon, C.J.F., Lugtenberg, D., Roosing, S., Bax, N.M., Blokland, E.A.W., et al. (2018). The Common ABCA4 Variant p.Asn1868Ile Shows Nonpenetrance and Variable Expression of Stargardt Disease When Present in trans With Severe Variants. Invest. Ophthalmol. Vis. Sci. *59*, 3220–3231.

10. Albert, S., Garanto, A., Sangermano, R., Khan, M., Bax, N.M., Hoyng, C.B., Zernant, J., Lee, W., Allikmets, R., Collin, R.W.J., and Cremers, F.P.M. (2018). Identification and Rescue of Splice Defects Caused by Two Neighboring Deep-Intronic ABCA4 Mutations Underlying Stargardt Disease. Am. J. Hum. Genet. *102*, 517–527.

11. Bauwens, M., De Zaeytijd, J., Weisschuh, N., Kohl, S., Meire, F., Dahan, K., Depasse, F., De Jaegere, S., De Ravel, T., De Rademaeker, M., et al. (2015). An augmented ABCA4 screen targeting noncoding regions reveals a deep intronic founder variant in Belgian Stargardt patients. Hum. Mutat. *36*, 39–42.

12. Bauwens, M., Garanto, A., Sangermano, R., Naessens, S., Weisschuh, N., De Zaeytijd, J., Khan, M., Sadler, F., Balikova, I., Van Cauwenbergh, C., et al. (2019). ABCA4-associated disease as a model for missing heritability in autosomal recessive disorders: novel noncoding splice, cis-regulatory, structural, and recurrent hypomorphic variants. Genet. Med. *21*, 1761–1771.

13. Bax, N.M., Sangermano, R., Roosing, S., Thiadens, A.A., Hoefsloot, L.H., van den Born, L.I., Phan, M., Klevering, B.J., Westeneng-van Haaften, C., Braun, T.A., et al. (2015). Heterozygous deep-intronic variants and deletions in ABCA4 in persons with retinal dystrophies and one exonic ABCA4 variant. Hum. Mutat. *36*, 43–47.

14. Sangermano, R., Garanto, A., Khan, M., Runhart, E.H., Bauwens, M., Bax, N.M., van den Born, L.I., Khan, M.I., Cornelis, S.S., Verheij, J.B.G.M., et al. (2019). Deep-intronic ABCA4 variants explain missing heritability in Stargardt disease and allow correction of splice defects by antisense oligonucleotides. Genet. Med. *21*, 1751–1760.

15. Schulz, H.L., Grassmann, F., Kellner, U., Spital, G., Rüther, K., Jägle, H., Hufendiek, K., Rating, P., Huchzermeyer, C., Baier, M.J., et al. (2017). Mutation Spectrum of the ABCA4 Gene in 335 Stargardt Disease Patients From a Multicenter German Cohort-Impact of Selected Deep Intronic Variants and Common SNPs. Invest. Ophthalmol. Vis. Sci. *58*, 394–403.

16. Zernant, J., Lee, W., Nagasaki, T., Collison, F.T., Fishman, G.A., Bertelsen, M., Rosenberg, T., Gouras, P., Tsang, S.H., and Allikmets, R. (2018). Extremely hypomorphic and severe deep intronic variants in the *ABCA4* locus result in varying Stargardt disease phenotypes. Cold Spring Harb. Mol. Case Stud. *4*, a002733.

17. Zhang, Z., Xin, D., Wang, P., Zhou, L., Hu, L., Kong, X., and Hurst, L.D. (2009). Noisy splicing, more than expression regulation, explains why some exons are subject to nonsense-mediated mRNA decay. BMC Biol. *7*, 23.

18. Caminsky, N., Mucaki, E.J., and Rogan, P.K. (2014). Interpretation of mRNA splicing mutations in genetic disease: review of the literature and guidelines for information-theoretical analysis. F1000Res. *3*, 282.

19. Braun, T.A., Mullins, R.F., Wagner, A.H., Andorf, J.L., Johnston, R.M., Bakall, B.B., Deluca, A.P., Fishman, G.A., Lam, B.L., Weleber, R.G., et al. (2013). Non-exomic and synonymous variants in ABCA4 are an important cause of Stargardt disease. Hum. Mol. Genet. *22*, 5136–5145.

20. Collin, R.W., and Garanto, A. (2017). Applications of antisense oligonucleotides for the treatment of inherited retinal diseases. Curr. Opin. Ophthalmol. *28*, 260–266.

21. Cideciyan, A.V., Jacobson, S.G., Drack, A.V., Ho, A.C., Charng, J., Garafalo, A.V., Roman, A.J., Sumaroka, A., Han, I.C., Hochstedler, M.D., et al. (2019). Effect of an intravitreal antisense oligonucleotide on vision in Leber congenital amaurosis due to a photoreceptor cilium defect. Nat. Med. *25*, 225–228.

22. Sangermano, R., Bax, N.M., Bauwens, M., van den Born, L.I., De Baere, E., Garanto, A., Collin, R.W., Goercharn-Ramlal, A.S., den Engelsman-van Dijk, A.H., Rohrschneider, K., et al. (2016). Photoreceptor Progenitor mRNA Analysis Reveals Exon Skipping Resulting from the ABCA4 c.5461-10T→C Mutation in Stargardt Disease. Ophthalmology *123*, 1375–1385.

23. Lois, N., Holder, G.E., Bunce, C., Fitzke, F.W., and Bird, A.C. (2001). Phenotypic subtypes of Stargardt macular dystrophy-fundus flavimaculatus. Arch. Ophthalmol. *119*, 359–369.

24. Sangermano, R., Khan, M., Cornelis, S.S., Richelle, V., Albert, S., Garanto, A., Elmelik, D., Qamar, R., Lugtenberg, D., van den Born, L.I., et al. (2018). *ABCA4* midigenes reveal the full splice spectrum of all reported noncanonical splice site variants in Stargardt disease. Genome Res. *28*, 100–110.

25. Parfitt, D.A., Lane, A., Ramsden, C.M., Carr, A.J., Munro, P.M., Jovanovic, K., Schwarz, N., Kanuga, N., Muthiah, M.N., Hull, S., et al. (2016). Identification and Correction of Mechanisms Underlying Inherited Blindness in Human iPSC-Derived Optic Cups. Cell Stem Cell *18*, 769–781.

26. Flamier, A., Barabino, A., and Gilbert, B. (2016). Differentiation of Human Embryonic Stem Cells into Cone Photoreceptors. Bio Protoc. *6*, e1870.

27. Dulla, K., Aguila, M., Lane, A., Jovanovic, K., Parfitt, D.A., Schulkens, I., Chan, H.L., Schmidt, I., Beumer, W., Vorthoren, L., et al. (2018). Splice-Modulating Oligonucleotide QR-110 Restores CEP290 mRNA and Function in Human c.2991+1655A>G LCA10 Models. Mol. Ther. Nucleic Acids *12*, 730–740.

28. Nakano, T., Ando, S., Takata, N., Kawada, M., Muguruma, K., Sekiguchi, K., Saito, K., Yonemura, S., Eiraku, M., and Sasai, Y. (2012). Self-formation of optic cups and storable stratified neural retina from human ESCs. Cell Stem Cell *10*, 771–785.

29. Parfitt, D.A., Lane, A., Ramsden, C., Jovanovic, K., Coffey, P.J., Hardcastle, A.J., and Cheetham, M.E. (2016). Using induced pluripotent stem cells to understand retinal ciliopathy disease mechanisms and develop therapies. Biochem. Soc. Trans. *44*, 1245–1251.

30. Zernant, J., Xie, Y.A., Ayuso, C., Riveiro-Alvarez, R., Lopez-Martinez, M.A., Simonelli, F., Testa, F., Gorin, M.B., Strom, S.P., Bertelsen, M., et al. (2014). Analysis of the ABCA4 genomic locus in Stargardt disease. Hum. Mol. Genet. *23*, 6797–6806.

31. Lee, Y., and Rio, D.C. (2015). Mechanisms and Regulation of Alternative Pre-mRNA Splicing. Annu. Rev. Biochem. *84*, 291–323.

32. Nassisi, M., Mohand-Saïd, S., Andrieu, C., Antonio, A., Condroyer, C., Méjécase, C., Varin, J., Wohlschlegel, J., Dhaenens, C.M., Sahel, J.A., et al. (2019). Prevalence of *ABCA4* Deep-Intronic Variants and Related Phenotype in An Unsolved "One-Hit" Cohort with Stargardt Disease. Int. J. Mol. Sci. *20*, 5053.

33. Runhart, E.H., Valkenburg, D., Cornelis, S.S., Khan, M., Sangermano, R., Albert, S., Bax, N.M., Astuti, G.D.N., Gilissen, C., Pott, J.R., et al. (2019). Late-Onset Stargardt Disease Due to Mild, Deep-Intronic ABCA4 Alleles. Invest. Ophthalmol. Vis. Sci. *60*, 4249–4256.

34. Zernant, J., Lee, W., Collison, F.T., Fishman, G.A., Sergeev, Y.V., Schuerch, K., Sparrow, J.R., Tsang, S.H., and Allikmets, R. (2017). Frequent hypomorphic alleles account for a significant fraction of ABCA4 disease and distinguish it from age-related macular degeneration. J. Med. Genet. *54*, 404–412.

35. Garanto, A., Duijkers, L., Tomkiewicz, T.Z., and Collin, R.W.J. (2019). Antisense Oligonucleotide Screening to Optimize the Rescue of the Splicing Defect Caused by the Recurrent Deep-Intronic *ABCA4* Variant c.4539+2001G>A in Stargardt Disease. Genes (Basel) *10*, 452.

36. Aartsma-Rus, A., van Vliet, L., Hirschi, M., Janson, A.A., Heemskerk, H., de Winter, C.L., de Kimpe, S., van Deutekom, J.C., 't Hoen, P.A., and van Ommen, G.J. (2009). Guidelines for antisense oligonucleotide design and insight into splice-modulating mechanisms. Mol. Ther. *17*, 548–553.

37. Zhou, L.Y., Qin, Z., Zhu, Y.H., He, Z.Y., and Xu, T. (2019). Current RNA-based Therapeutics in Clinical Trials. Curr. Gene Ther. *19*, 172–196.

38. Garanto, A., Chung, D.C., Duijkers, L., Corral-Serrano, J.C., Messchaert, M., Xiao, R., Bennett, J., Vandenberghe, L.H., and Collin, R.W. (2016). In vitro and in vivo rescue of aberrant splicing in CEP290-associated LCA by antisense oligonucleotide delivery. Hum. Mol. Genet. *25*, 2552–2563.

39. Goyenvalle, A., Vulin, A., Fougerousse, F., Leturcq, F., Kaplan, J.C., Garcia, L., and Danos, O. (2004). Rescue of dystrophic muscle through U7 snRNA-mediated exon skipping. Science *306*, 1796–1799.

40. Carss, K.J., Arno, G., Erwood, M., Stephens, J., Sanchis-Juan, A., Hull, S., Megy, K., Grozeva, D., Dewhurst, E., Malka, S., et al.; NIHR-BioResource Rare Diseases Consortium (2017). Comprehensive Rare Variant Analysis via Whole-Genome Sequencing to Determine the Molecular Pathology of Inherited Retinal Disease. Am. J. Hum. Genet. *100*, 75–90.

41. Jaakson, K., Zernant, J., Külm, M., Hutchinson, A., Tonisson, N., Glavac, D., Ravnik-Glavac, M., Hawlina, M., Meltzer, M.R., Caruso, R.C., et al. (2003). Genotyping microarray (gene chip) for the ABCR (ABCA4) gene. Hum. Mutat. *22*, 395–403.

42. McCulloch, D.L., Marmor, M.F., Brigell, M.G., Hamilton, R., Holder, G.E., Tzekov, R., and Bach, M. (2015). ISCEV Standard for full-field clinical electroretinography (2015 update). Doc. Ophthalmol. *130*, 1–12.

43. Desmet, F.O., Hamroun, D., Lalande, M., Collod-Béroud, G., Claustres, M., and Béroud, C. (2009). Human Splicing Finder: an online bioinformatics tool to predict splicing signals. Nucleic Acids Res. *37*, e67.

44. Pertea, M., Lin, X., and Salzberg, S.L. (2001). GeneSplicer: a new computational method for splice site prediction. Nucleic Acids Res. *29*, 1185–1190.

45. Reese, M.G., Eeckman, F.H., Kulp, D., and Haussler, D. (1997). Improved splice site detection in Genie. J. Comput. Biol. *4*, 311–323.

46. Shapiro, M.B., and Senapathy, P. (1987). RNA splice junctions of different classes of eukaryotes: sequence statistics and functional implications in gene expression. Nucleic Acids Res. *15*, 7155–7174.

47. Yeo, G., and Burge, C.B. (2004). Maximum entropy modeling of short sequence motifs with applications to RNA splicing signals. J. Comput. Biol. *11*, 377–394.

48. Aartsma-Rus, A. (2012). Overview on AON design. Methods Mol. Biol. *867*, 117–129.

49. Garanto, A., and Collin, R.W.J. (2018). Design and In Vitro Use of Antisense Oligonucleotides to Correct Pre-mRNA Splicing Defects in Inherited Retinal Dystrophies. Methods Mol. Biol. *1715*, 61–78.

50. Takahashi, K., and Yamanaka, S. (2006). Induction of pluripotent stem cells from mouse embryonic and adult fibroblast cultures by defined factors. Cell *126*, 663–676.

51. Schindelin, J., Arganda-Carreras, I., Frise, E., Kaynig, V., Longair, M., Pietzsch, T., Preibisch, S., Rueden, C., Saalfeld, S., Schmid, B., et al. (2012). Fiji: an open-source platform for biological-image analysis. Nat. Methods *9*, 676–682.

52. Okita, K. (2011). iPS cells for transplantation. Curr. Opin. Organ Transplant. *16*, 96–100.

OMTN, Volume *21*

Supplemental Information

Detailed Phenotyping and Therapeutic Strategies for Intronic *ABCA4* Variants in Stargardt Disease

Mubeen Khan, Gavin Arno, Ana Fakin, David A. Parfitt, Patty P.A. Dhooge, Silvia Albert, Nathalie M. Bax, Lonneke Duijkers, Michael Niblock, Kwan L. Hau, Edward Bloch, Elena R. Schiff, Davide Piccolo, Michael C. Hogden, Carel B. Hoyng, Andrew R. Webster, Frans P.M. Cremers, Michael E. Cheetham, Alejandro Garanto, and Rob W.J. Collin

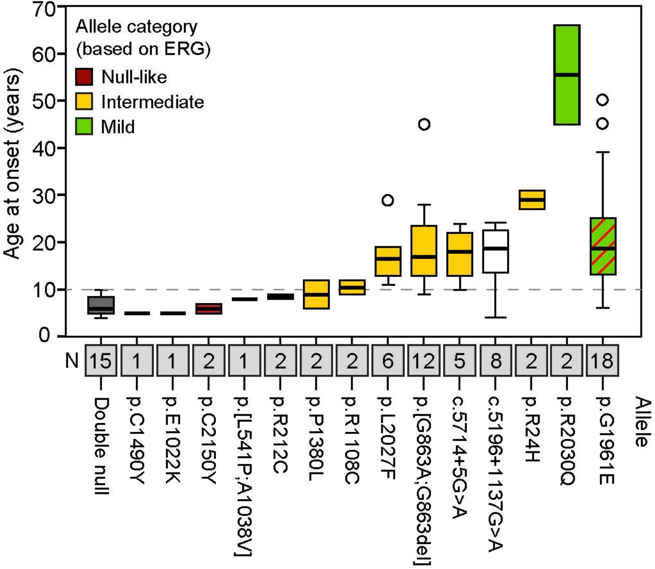
Allele category
(based on ERG)
Null-like
Intermediate
Mild
Age at onset (years)
0
10
20
30
40
50
60
70
N
15
1
1
2
1
2
2
2
6
12
5
8
2
2
18
Double null
p.C1490Y
p.E1022K
p.C2150Y
p.[L541P;A1038V]
p.R212C
p.P1380L
p.R1108C
p.L2027F
p.[G863A;G863del]
c.5714+5G>A
c.5196+1137G>A
p.R24H
p.R2030Q
p.G1961E
Allele

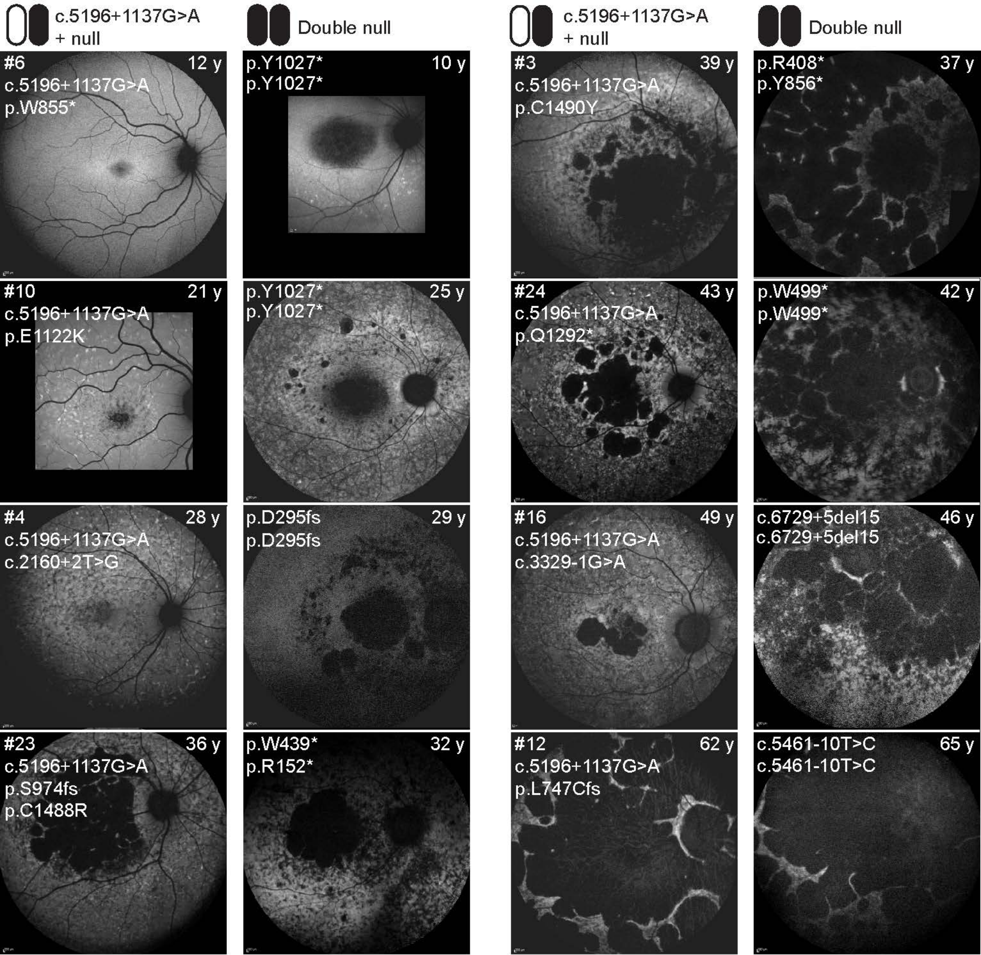
c.5196+1137G>A
+ null
Double null
c.5196+1137G>A
+ null
Double null
#6 12 y
c.5196+1137G>A
p.W855*
p.Y1027* 10 y
p.Y1027*
#3 39 y
c.5196+1137G>A
p.C1490Y
p.R408* 37 y
p.Y856*
#10 21 y
c.5196+1137G>A
p.E1122K
p.Y1027* 25 y
p.Y1027*
#24 43 y
c.5196+1137G>A
p.Q1292*
p.W499* 42 y
p.W499*
#4 28 y
c.5196+1137G>A
c.2160+2T>G
p.D295fs 29 y
p.D295fs
#16 49 y
c.5196+1137G>A
c.3329-1G>A
c.6729+5del15 46 y
c.6729+5del15
#23 36 y
c.5196+1137G>A
p.S974fs
p.C1488R
p.W439* 32 y
p.R152*
#12 62 y
c.5196+1137G>A
p.L747Cfs
c.5461-10T>C 65 y
c.5461-10T>C

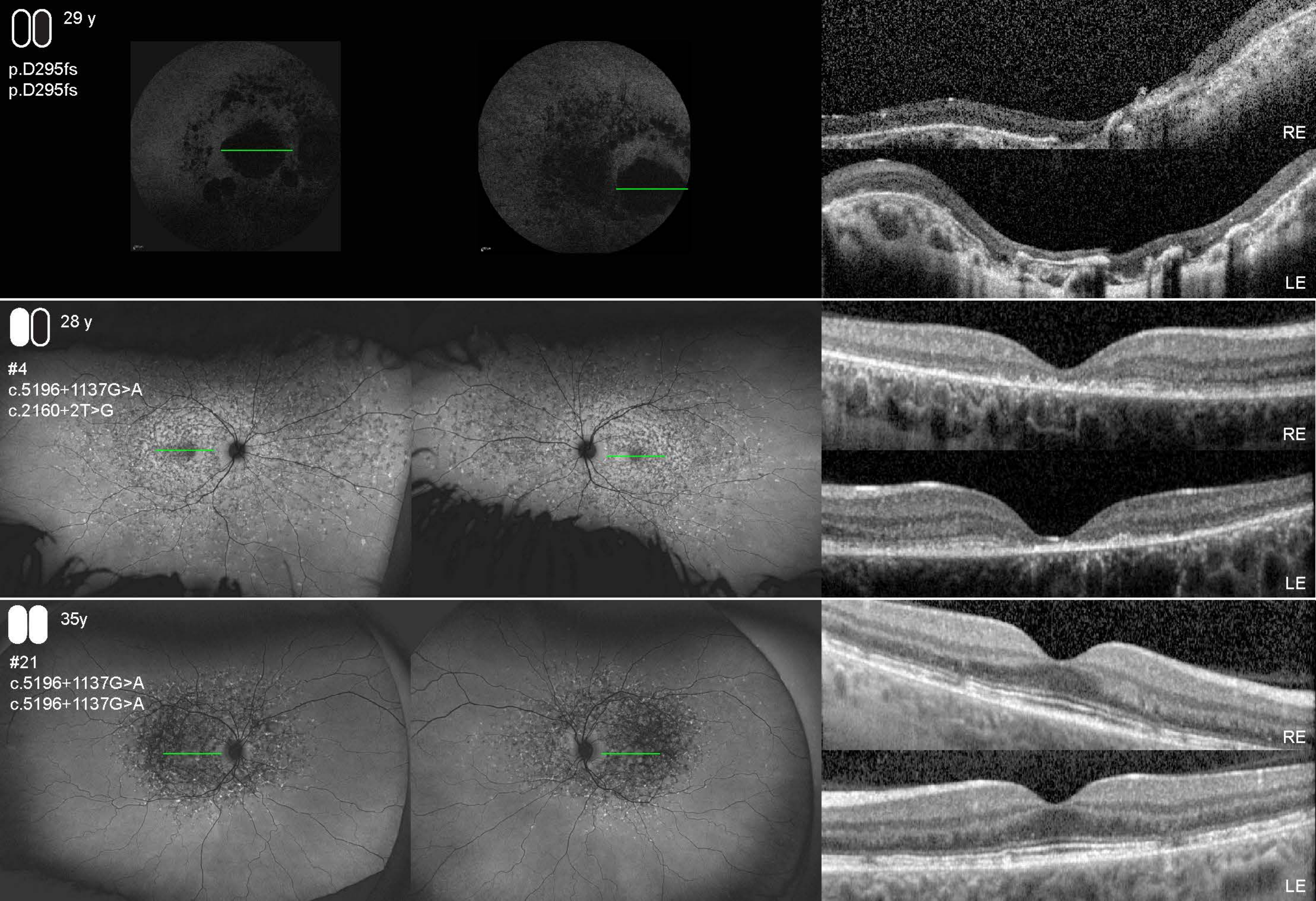
29 y
p.D295fs
p.D295fs
RE
LE
28 y
#4
c.5196+1137G>A
c.2160+2T>G
RE
LE
35y
#21
c.5196+1137G>A
c.5196+1137G>A
RE
LE

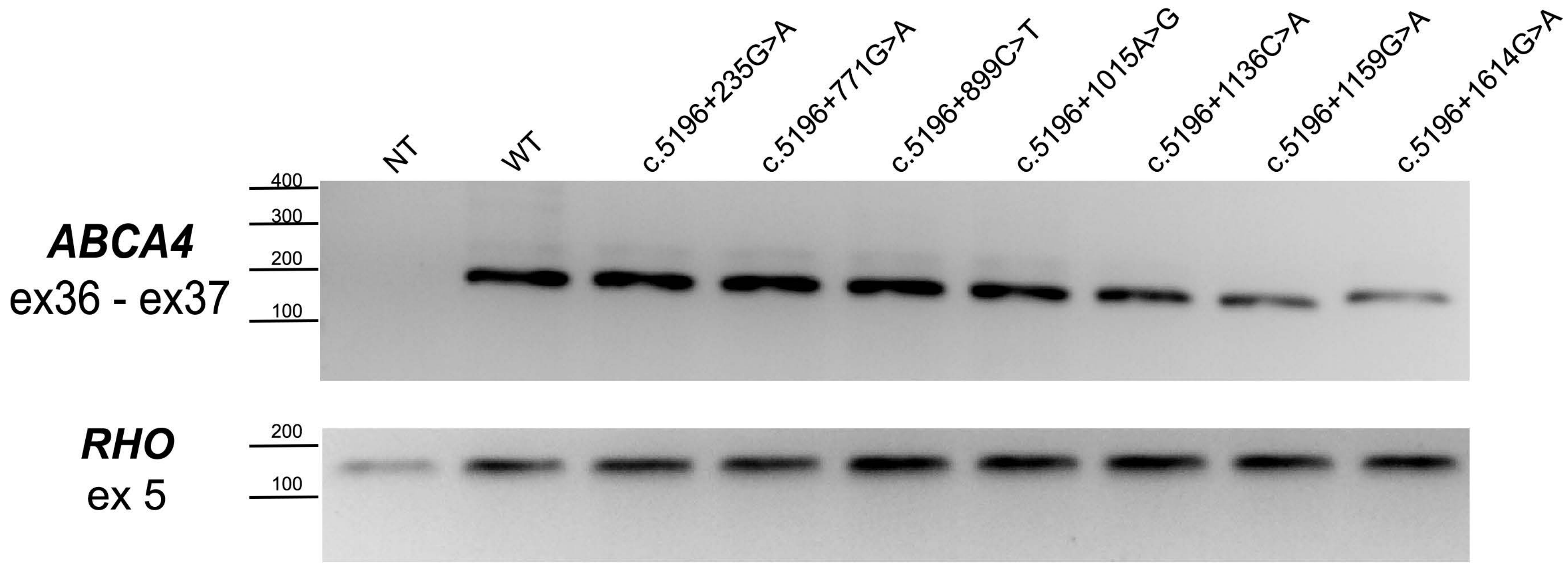
NT
WT
c.5196+235G>A
c.5196+771G>A
c.5196+899C>T
c.5196+1015A>G
c.5196+1136C>A
c.5196+1159G>A
c.5196+1614G>A
400
300
200
100
ABCA4
ex36 - ex37
RHO
ex 5
200
100

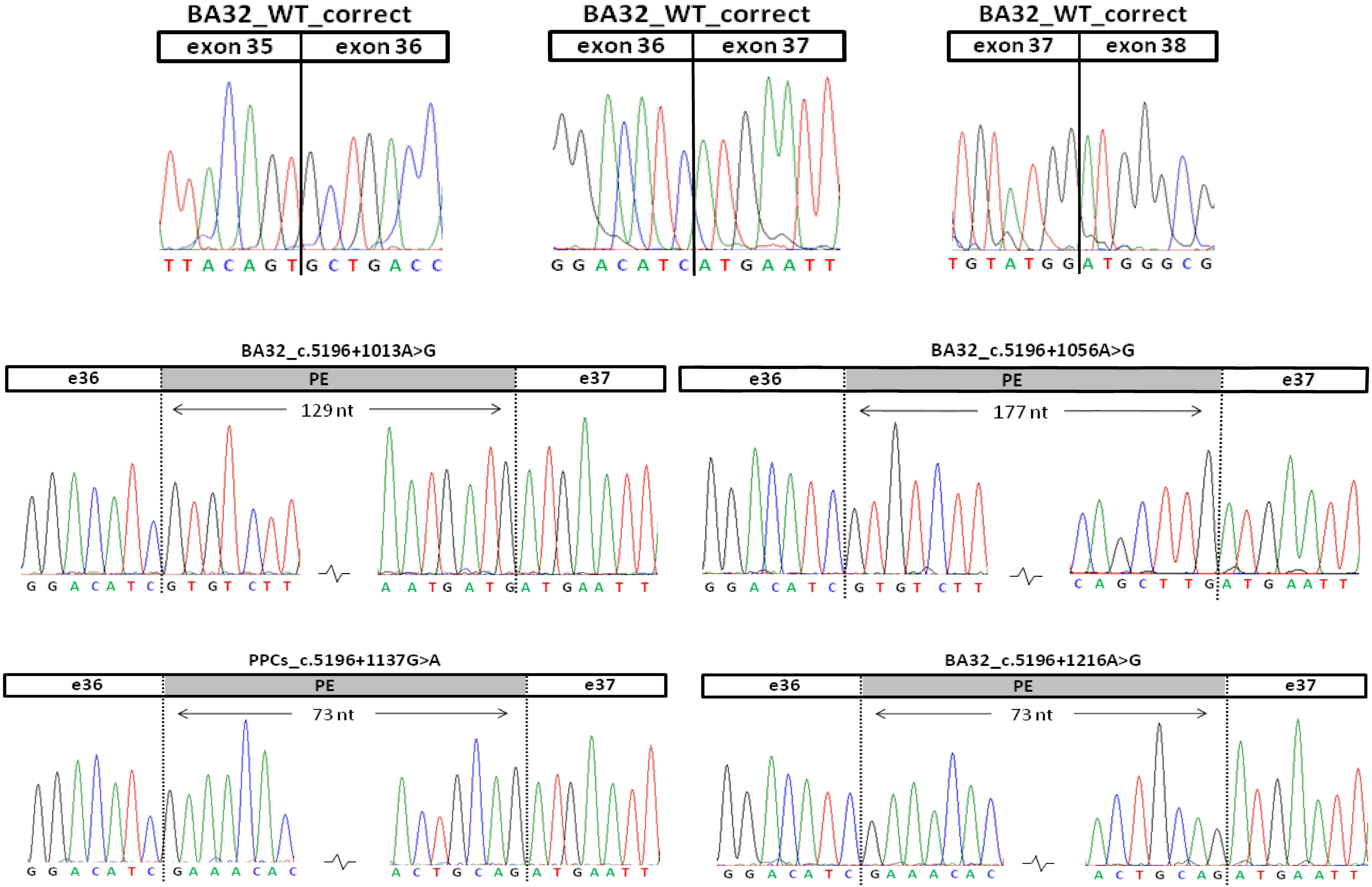
BA32_WT_correct
exon 35
exon 36
T T A C A G T G C T G A C C
BA32_WT_correct
exon 36
exon 37
G G A C A T C A T G A A T T
BA32_WT_correct
exon 37
exon 38
T G T A T G G A T G G G C G
BA32_c.5196+1013A>G
e36
PE
e37
129 nt
G G A C A T C G T G T C T T
A A T G A T G A T G A A T T
BA32_c.5196+1056A>G
e36
PE
e37
177 nt
G G A C A T C G T G T C T T
C A G C T T G A T G A A T T
PPCs_c.5196+1137G>A
e36
PE
e37
73 nt
G G A C A T C G A A A C A C
A C T G C A G A T G A A T T
BA32_c.5196+1216A>G
e36
PE
e37
73 nt
G G A C A T C G A A A C A C
A C T G C A G A T G A A T T

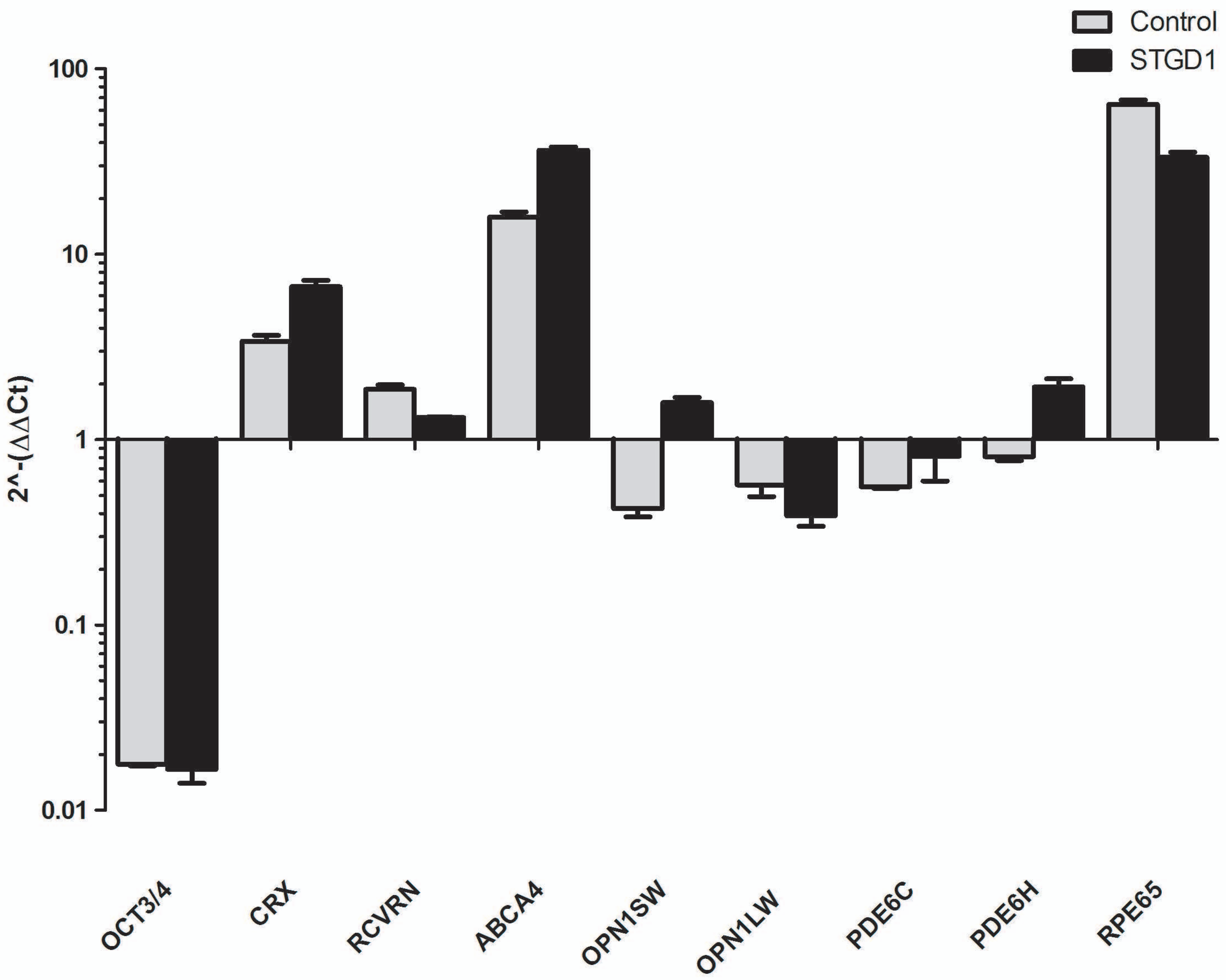

Control
STGD1
100
10
1
0.1
0.01
2^-(ΔΔCt)
OCT3/4
CRX
RCVRN
ABCA4
OPN1SW
OPN1LW
PDE6C
PDE6H
RPE65

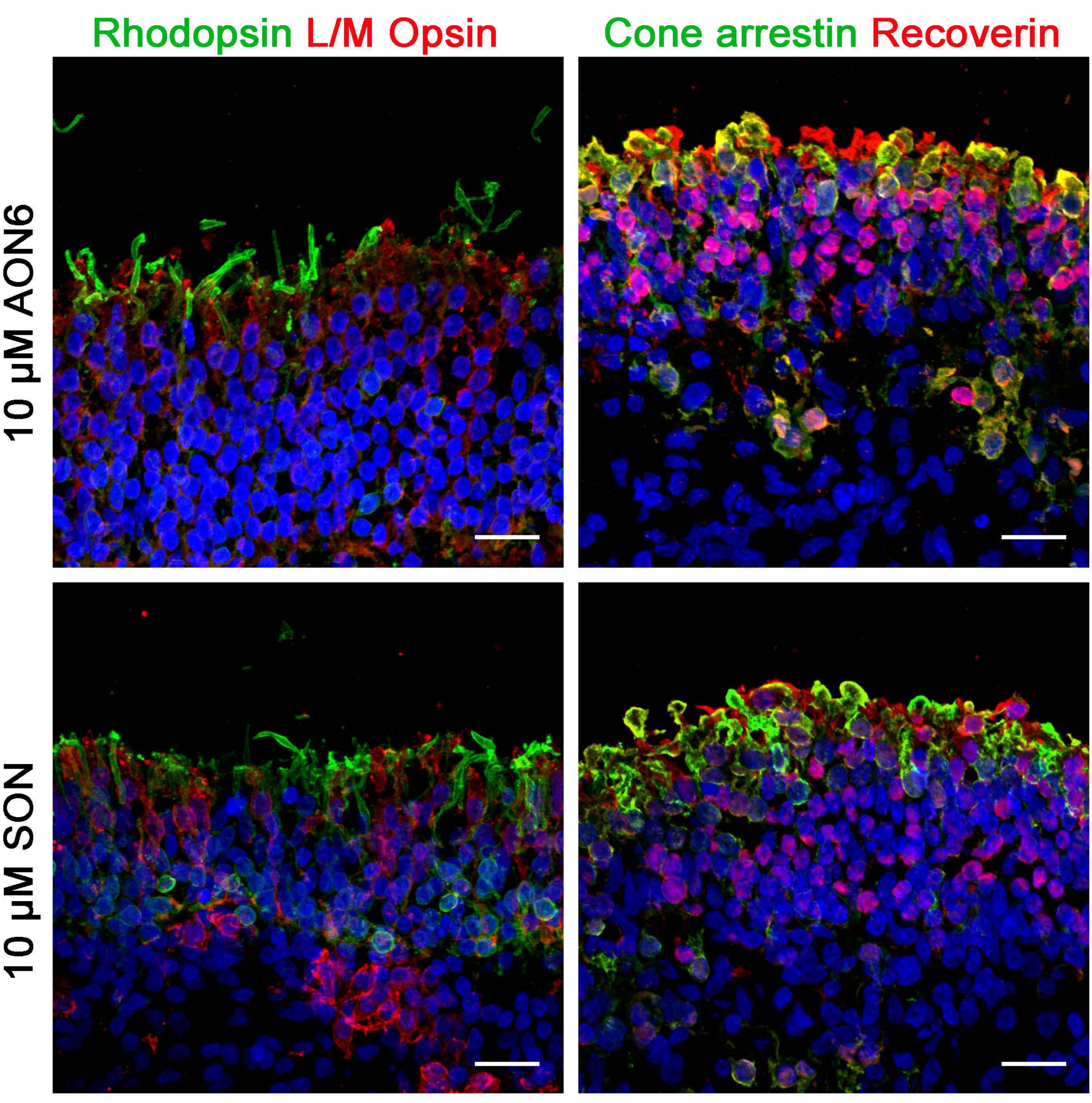

Rhodopsin L/M Opsin
Cone arrestin Recoverin
10 μM AON6
10 μM SON

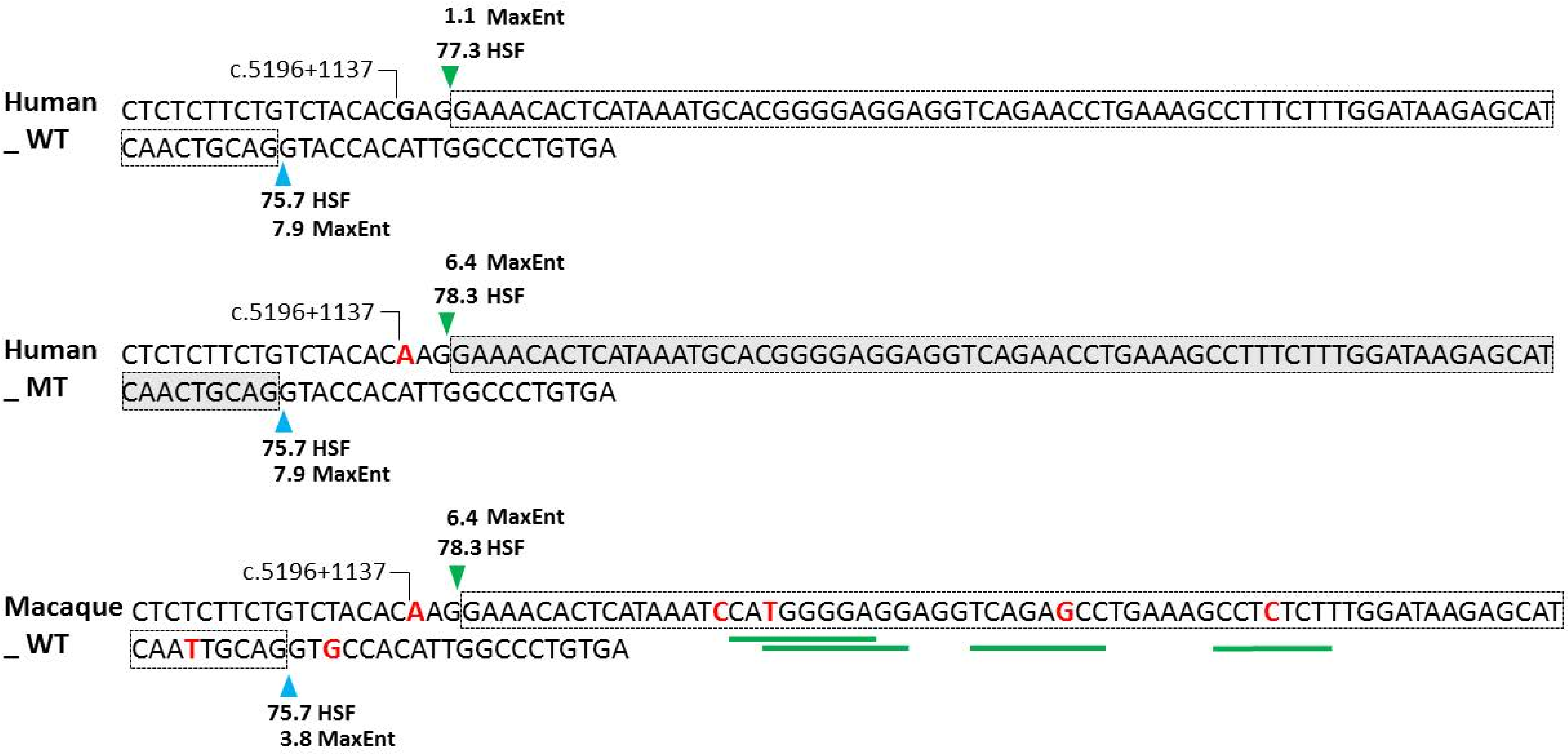
1.1 MaxEnt
77.3 HSF
c.5196+1137
Human _WT
CTCTCTTCTGTCTACACGAGGAAACACTCATAAATGCACGGGGAGGAGGTCAGAACCTGAAAGCCTTTCTTTGGATAAGAGCAT
CAACTGCAGGTACCACATTGGCCCTGTGA
75.7 HSF
7.9 MaxEnt
6.4 MaxEnt
78.3 HSF
c.5196+1137
Human _MT
CTCTCTTCTGTCTACACAAGGAAACACTCATAAATGCACGGGGAGGAGGTCAGAACCTGAAAGCCTTTCTTTGGATAAGAGCAT
CAACTGCAGGTACCACATTGGCCCTGTGA
75.7 HSF
7.9 MaxEnt
6.4 MaxEnt
78.3 HSF
c.5196+1137
Macaque _WT
CTCTCTTCTGTCTACACAAGGAAACACTCATAAATCCATGGGGAGGAGGTCAGAGCCTGAAAGCCTCTCTTTGGATAAGAGCAT
CAATTGCAGGTGCCACATTGGCCCTGTGA
75.7 HSF
3.8 MaxEnt

Supplemental Figure Legends

Supplemental Figure S1

Distribution of the ages at onset associated with different *ABCA4* alleles in *trans* with null alleles. The figure is modified from Fakin et al,[7] with the addition of c.5196+1137G>A. Different categories of alleles were determined based on full-field ERG. Allele p.G1961E had a specific preponderance to foveal damage, reflected by a relatively early onset of visual symptoms. Dashed line marks the 95% confidence interval of the patients harboring two null alleles.

Supplemental Figure S2

Fundus autofluorescence images of patients harboring c.5196+1137G>A in *trans* with null alleles and age-matched patients harboring two null alleles. Patient ID and genotypes are noted in the top left corner; age is noted in the top right corner of each image.

Supplemental Figure S3

FAF and OCT of a patient harboring two null alleles (top), a patient harboring c.5196+1137G>A in *trans* with a null allele (middle) and a patient homozygous for c.5196+1137G>A patient of similar ages. Note the increasingly milder phenotype in the presence of c.5196+1137G>A alleles.

Supplemental Figure S4

Sanger sequencing electropherograms for four causal *ABCA4* variants in intron 36 and correct transcript are given. Sequence traces for variants tested in *in vitro* splice assays are labeled as BA32_respective variant, whereas results obtained from photoreceptor progenitor cells (PPCs) are labelled as PPCs variants. PE, pseudoexon; nt, nucleotide.

Supplemental Figure S5

Seven *ABCA4* variants in intron 36 were tested in wild-type (WT) construct BA32 (*ABCA4* exons 35 - exon 38). RT-PCR was performed by using exonic (ex) primers in exon 36 and 37 of *ABCA4* for WT and all the variants. Rhodopsin (*RHO*) exon 5 amplification was used as a transfection and loading control.

Supplemental Figure S6

Gene expression profile of photoreceptor precursor cells (PPCs) derived from control (grey bars) and c.5196+1137G>A STGD1 (black bars) iPSCs. Expression levels were assessed by qPCR, normalized to GUSB expression and compared to the expression profile of day 0 iPSCs (undifferentiated). Pluripotency marker expression (*OCT3/4*) was reduced, while the expression of the photoreceptor precursor marker (*CRX*) was increased. The differentiation into RPE-like cells is shown by the increased expression of *RPE65*, while PPCs (especially STGD1 cells) showed some expression of early (*RCVRN*, *OPN1SW*) and late (*OPN1LW, PDE6C or PDE6H*). The expression of *ABCA4* was highly increased in all cell lines. The results are shown as the mean ± SD of two experimental replicates with three technical replicates each.

Supplemental Figure S7

Immunocytochemistry of retinal organoids treated with either AON or SON. Sections were stained with antibodies for rhodopsin (green, left panels), L/M opsin (red, left panels), cone arrestin (green, right panels) and recoverin (red, right panels). Nuclei were stained with DAPI in blue. Scale bar: 20 µM.

Supplemental Figure S8

Splice-site predictions are given for human wild type (WT) and mutant (MT) sequence as well as for the macaque WT. Strength of the WT and MT splice acceptor site (SAS) and splice donor site (SDS) were calculated using Human splicing Finder (HSF) and MaxEnt splicing prediction tools, by taking 20 nt upstream and downstream of the 73-nt pseudo-exon (PE). The PE found in the human mutant sequence is highlighted in grey. Nucleotides that are different among human WT/MT and macaque WT are shown in red. Green bars indicate the newly created splicing silencer elements due to the difference in human and macaque sequences. Green and blue rectangles indicate the SAS and SDS respectively.
